# The Eph receptor A4 plays a role in demyelination and depression-related behavior

**DOI:** 10.1172/JCI152187

**Published:** 2022-04-15

**Authors:** Yuan Li, Ping Su, Yuxiang Chen, Jing Nie, Ti-Fei Yuan, Albert H.C. Wong, Fang Liu

**Affiliations:** 1 Shanghai Mental Health Center, Shanghai Jiaotong University, School of Medicine, Shanghai, China.; 2 Campbell Family Mental Health Research Institute, Centre for Addiction and Mental Health,; 3 Departments of Psychiatry,; 4 Institutes of Medical Science,; 5 Pharmacology and Toxicology, and; 6 Physiology at the University of Toronto, Toronto, Ontario, Canada.

**Keywords:** Cell Biology, Neuroscience, Demyelinating disorders, Molecular biology

## Abstract

Proper myelination of axons is crucial for normal sensory, motor, and cognitive function. Abnormal myelination is seen in brain disorders such as major depressive disorder (MDD), but the molecular mechanisms connecting demyelination with the pathobiology remain largely unknown. We observed demyelination and synaptic deficits in mice exposed to either chronic, unpredictable mild stress (CUMS) or LPS, 2 paradigms for inducing depression-like states. Pharmacological restoration of myelination normalized both synaptic deficits and depression-related behaviors. Furthermore, we found increased ephrin A4 receptor (EphA4) expression in the excitatory neurons of mice subjected to CUMS, and shRNA knockdown of EphA4 prevented demyelination and depression-like behaviors. These animal data are consistent with the decrease in myelin basic protein and the increase in EphA4 levels we observed in postmortem brain samples from patients with MDD. Our results provide insights into the etiology of depressive symptoms in some patients and suggest that inhibition of EphA4 or the promotion of myelination could be a promising strategy for treating depression.

## Graphical abstract

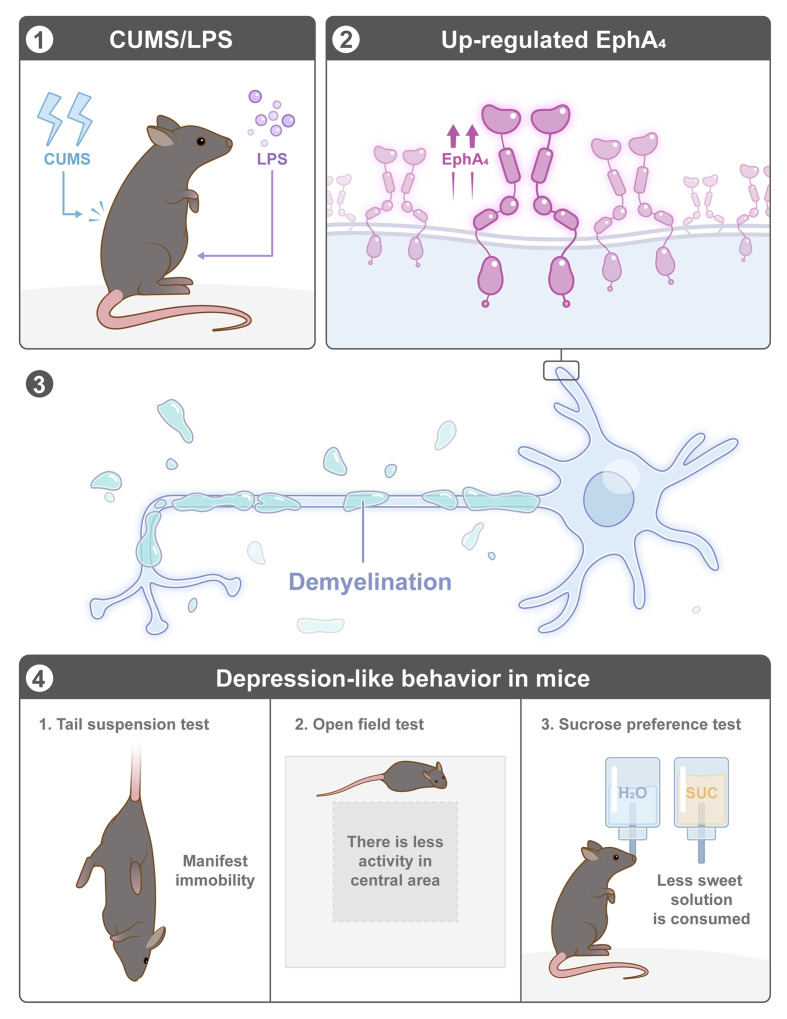

## Introduction

Major depressive disorder (MDD) is a very common and severe psychiatric disorder that affects approximately 300 million people worldwide (1), with a mortality rate of up to 6%, usually by suicide (2). The core feature of MDD is depressed mood, accompanied by disturbed sleep, memory, and concentration, accompanied by low energy, motivation, libido, and appetite (3). Many patients are refractory to current antidepressant medications (4, 5), which, despite significant improvements over older drugs, can still cause intolerable side effects (6, 7). Many modalities of psychotherapy are also effective, but these require substantial resources and are also ineffective for many patients (8). Electroconvulsive therapy is an old but highly effective treatment for depression that has very significant side effects (9), and emerging treatments such as ketamine (10) and other brain stimulation techniques require further validation (11, 12).

This large variety of treatments parallels the heterogenous neurobiology of depression, which has a plethora of neural correlates and putative causes spanning the full range of the biopsychosocial model (13). In the current study, we focused on investigating demyelination as one pathobiological mechanism for MDD (14–16). Emerging in vivo neuroimaging studies have shown that patients with MDD have abnormal white matter and myelin structure in multiple brain regions, including the dorsolateral prefrontal cortex, the anterior cingulate cortex, the hippocampus, and the corpus callosum (17–19). Consistent with these findings, postmortem brain tissue from patients with MDD has decreased oligodendrocyte density and oligodendrocyte-related gene expression (1, 20). Conversely, there is a high prevalence of depression in patients with multiple sclerosis (MS), an autoimmune demyelinating disorder (21). Animal studies provide further support for this link between demyelination, oligodendrocyte dysfunction, and depression-related behaviors (22, 23). In particular, focal demyelination in the medial prefrontal cortex (mPFC) is sufficient to induce behaviors relevant to depression in mice (24–26).

The ephrin A4 receptor (EphA4) is a member of the Eph family of receptor tyrosine kinases (RTKs). Activation of EphA4 decreases myelination in both the CNS and PNS (27–29). EphA4 has also been shown to modulate synaptic plasticity (30), axon guidance (31), and neurogenesis (32), and all of these neural functions have previously been implicated in the pathobiology of depression (33, 34). However, direct evidence demonstrating the involvement of EphA4 in depression has not been published.

In this study, we investigated how myelination might affect the cellular and behavioral correlates of depression in animal models and in postmortem human brain samples from patients with MDD. We used 2 common paradigms for inducing depression-like states: chronic, unpredictable mild stress (CUMS) and LPS. There are many variations of CUMS described in the literature, but the principle is to expose animals to a varied spectrum of stressful but not physically harmful environmental insults that change from day to day, such as noise, dampness, food and water deprivation, or light-dark cycle reversal (7, 35). LPS is a bacterial constituent that causes inflammation when injected into animals; repeated exposure can induce depression-like states (7, 15).

We hypothesized that inducing depression-like states in mice through environmental insults would cause demyelination and that increased EphA4 expression is necessary to mediate these cellular effects. Furthermore, we hypothesized that pharmacological enhancement of myelination or knocking down EphA4 would reverse or prevent these pathological changes, respectively. We used CUMS and LPS to induce depression-like states and clemastine for pharmacological rescue. Similarly, we predicted that demyelination and upregulation of EphA4 would be observed in brain tissue from patients with MDD.

Our experiments demonstrate that EphA4 is necessary for the demyelination and synaptic deficits seen in animal models for the study of depression, and this observation is supported by convergent observational data from human brain tissue samples. We identify a molecular mechanism by which environmental stressors can be transduced into structural changes in the CNS, which is a longstanding paradigm for explaining the origins of mental disorders, especially depression. Our work also provides the basis for new approaches to treating depression, such as by specifically inhibiting EphA4 or by promoting myelination in general as a target for future therapeutics.

## Results

### Demyelination in mouse models relevant to depression.

**Figure 1:**
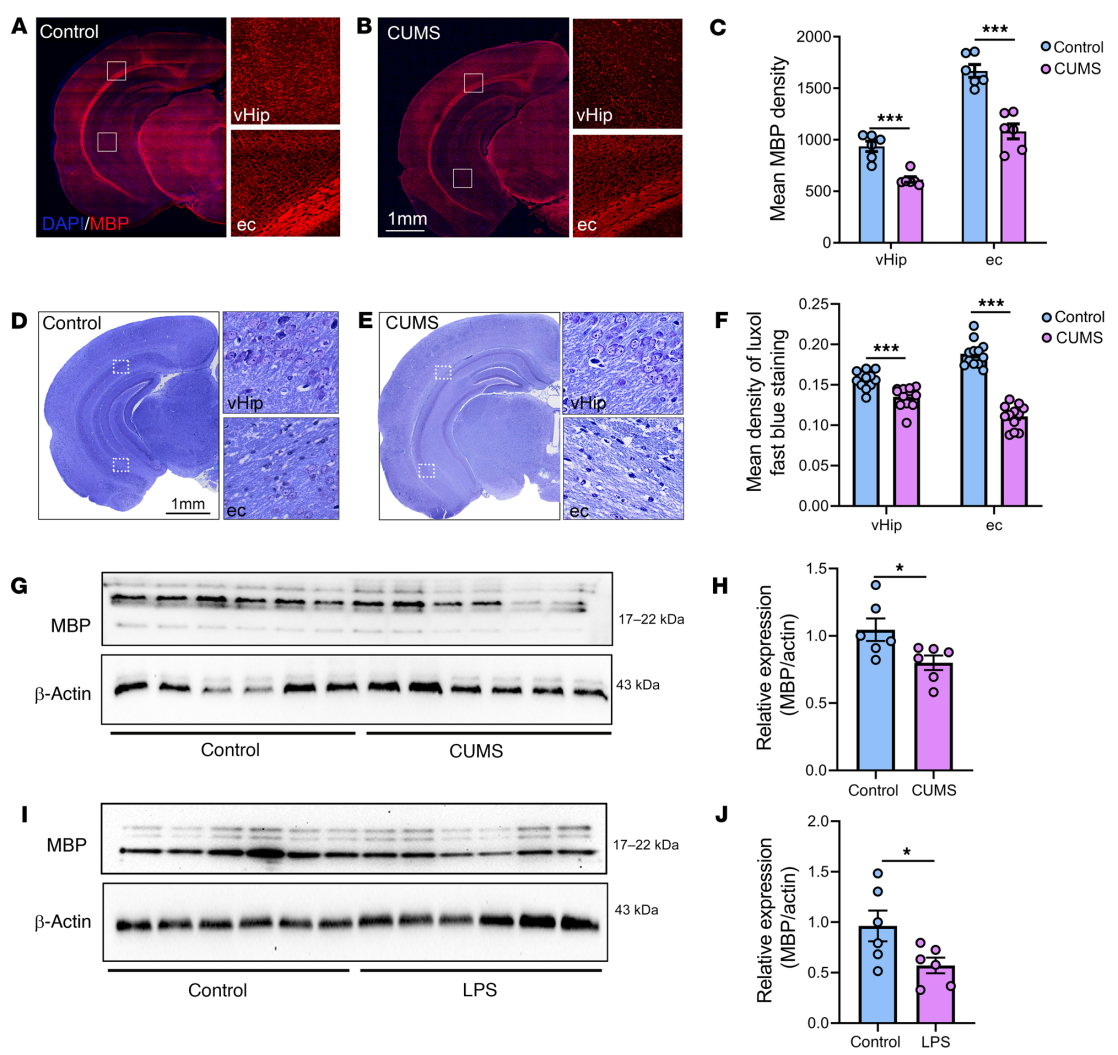
Decreased myelination was observed in mouse models for the study of depression. (**A** and **B**) Representative immunofluorescence images of MBP expression in brain sections from control (**A**) and CUMS (**B**) mice. Panels on the right are higher-magnification images of the ventral hippocampus (vHip) and external capsule (ec). Scale bar: 1 mm; original magnification, ×100 (enlarged insets). (**C**) Quantification of MBP fluorescence intensity in the ventral hippocampus (*t*_10_ = 5.681) and the external capsule (*t*_10_ = 6.130). *n* = 6 slices from 3 animal brains/group. (**D** and **E**) Representative images of LFB histological staining. Scale bar: 1 mm; original magnification, ×200 (enlarged insets). (**F**) Quantification results of LFB staining of the ventral hippocampus (*t*_22_ = 4.410) and the external capsule (*t*_22_ = 12.40). *n* = 12 slices from 4 animal brains/group. (**G**–**J**) Western blots and analysis showing lower MBP expression in ventral hippocampus from CUMS mice (**G** and **H**) (*t*_10_ = 2.446, *n* = 6 brains/group) and LPS-treated mice (**I** and **J**) (*t*_10_ = 2.291, *n* = 6 brains/group) mice. β-Actin was used as the loading control. Data are shown as the mean ± SEM. *P < 0.05 and ***P < 0.001, by unpaired Student’s *t* test (**C**, **F**, **H**, and **J**).

We first examined the CUMS and LPS mouse models for studying depression to confirm the presence of demyelination and depression-related behavior. CUMS was applied as previously described (7, 35). As shown in Supplemental Figure 1, A–H; supplemental material available online with this article; https://doi.org/10.1172/JCI152187DS1, we conducted 3 common behavioral tests related to depression: the sucrose preference test (SPT), the open field test (OFT), and the tail suspension test (TST) on day 42 of the CUMS protocol, and we performed immunohistochemical analysis using antibodies against myelin basic protein (MBP), the most common cellular marker for myelin. We found significantly decreased MBP levels in the hippocampus of mice subjected to CUMS (referred to hereafter as CUMS mice) compared with the levels in control animals (Figure 1, A–C). We also used Luxol fast blue (LFB) staining to confirm reduced myelination in the CUMS mouse brain (Figure 1, D–F). Western blot analysis of protein extracted from hippocampus produced results consistent with our histopathology results (Figure 1, G and H). In addition, we measured MBP expression in striatum and hypothalamus by Western blotting and found decreased MBP protein levels in striatum but not hypothalamus (Supplemental Figure 2, A–D). Western blotting also revealed a similar decrease in MBP expression in the hippocampus of the LPS mouse model (Figure 1, I and J).

**Figure 2:**
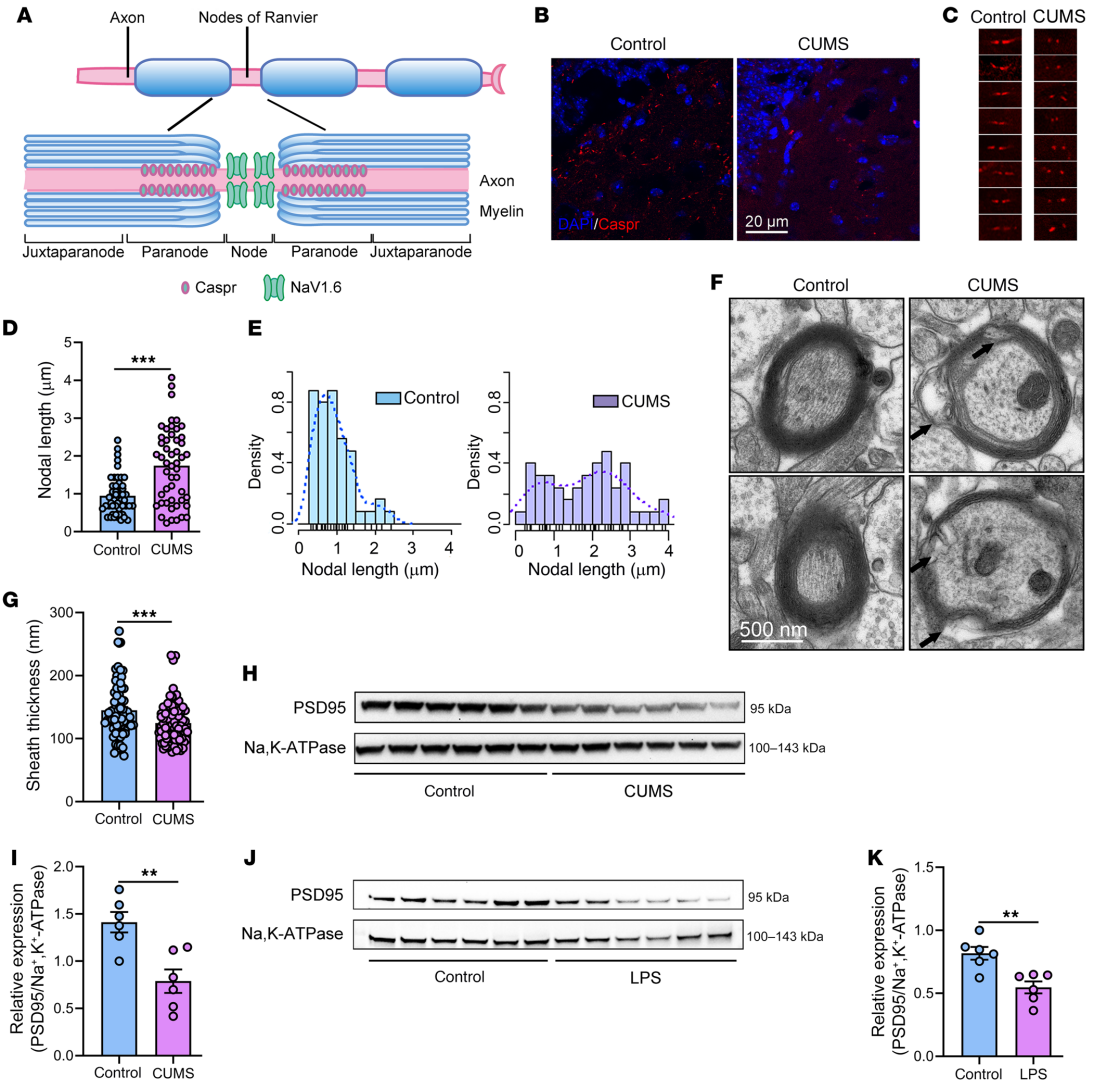
Demyelination and altered synaptic protein expression are observed in mouse models relevant to depression. (**A**) Schematic diagram of the myelin sheath, showing the nodes of Ranvier, the paranode, and the juxtaparanode, with Caspr 1 expressed mainly in the paranode. (**B**) Representative images showing Caspr-positive, red-stained paranodal regions in the ventral hippocampus. Scale bar: 20 μm. *n* = 4 mice/group. (**C**) High-magnification images of Caspr staining from **B**. Original magnification, ×400. (**D**) Nodal lengths were increased in CUMS mice, based on measurements of Caspr-stained regions (*n* = 50 nodes from 3 different mice/group). (**E**) Histograms showing the frequency distribution of nodal length, which differed between control and CUMS mice. (**F**) Representative electron microscopic images showing demyelination in CUMS mice. Scale bar: 500 nm. (**G**) Thinner myelin sheaths were observed in CUMS mice, as measured by electron microscopy. The total number of myelin sheaths analyzed in the control and CUMS groups was 73 and 87, respectively (*n* = 15 images from 5 mice/group, *t*_158_ = 3.361). (**H** and **I**) Representative blots showing decreased PSD95 protein expression in CUMS mice and results of the densitometric analysis. Na^+^K^+^ATPase was used as the loading control (*n* = 6 mice/group, *t*_10_ = 3.798). (**J** and **K**) Representative blots showing decreased PSD95 protein expression in LPS-treated mice and results of the densitometric analysis (*n* = 6 mice/group, *t*_10_ = 3.866). Data are shown as the mean ± SEM. **P < 0.01 and ***P < 0.001, by unpaired Student’s *t* test (**D**, **G**, **I**, and **K**).

We then performed a more detailed histological analysis of the nodes of Ranvier on myelinated axons. Antibodies against contactin-associated protein (Caspr) were used to examine the paranodal junction in the hippocampus, and nodal length was measured according to the borders of Caspr-positive regions (Figure 2, A–D). Similar to previous studies in MS, in which demyelination increases nodal length (36), the mean nodal length in CUMS mice (1.74 ± 0.99 μm) was significantly longer than that in control mice (0.95 ± 0.50 μm). We also found an increase in the nodal length in corpus callosum (Supplemental Figure 3, A–D). Moreover, the distribution of nodal length in CUMS mice was broader (Figure 2E), and the axonal myelin sheath as visualized with electron microscopy was thinner compared with that of control animals (Figure 2, F and G). We also observed a decreased axonal myelin sheath in the corpus callosum (Supplemental Figure 3, E and F). These data demonstrate an association between demyelination and depression-related behaviors in the mouse.

### Altered synaptic protein expression in mouse models with features of depression.

Chronic stress has been reported to disrupt local protein synthesis in synapses, resulting in altered production of proteins required for the formation, maturation, and function of synapses (37). Thus, we measured the amount of postsynaptic density protein 95 (PSD95) in the membrane fraction of protein extracted from the hippocampus of depression-like model mice. We found that PSD95 was significantly decreased in both CUMS mice (Figure 2, H and I) and LPS mice (Figure 2, J and K). These data suggest altered synaptic composition in conjunction with demyelination and depression-like behaviors.

### Clemastine promotes myelination and rescues depression-related behaviors in mice.

**Figure 3:**
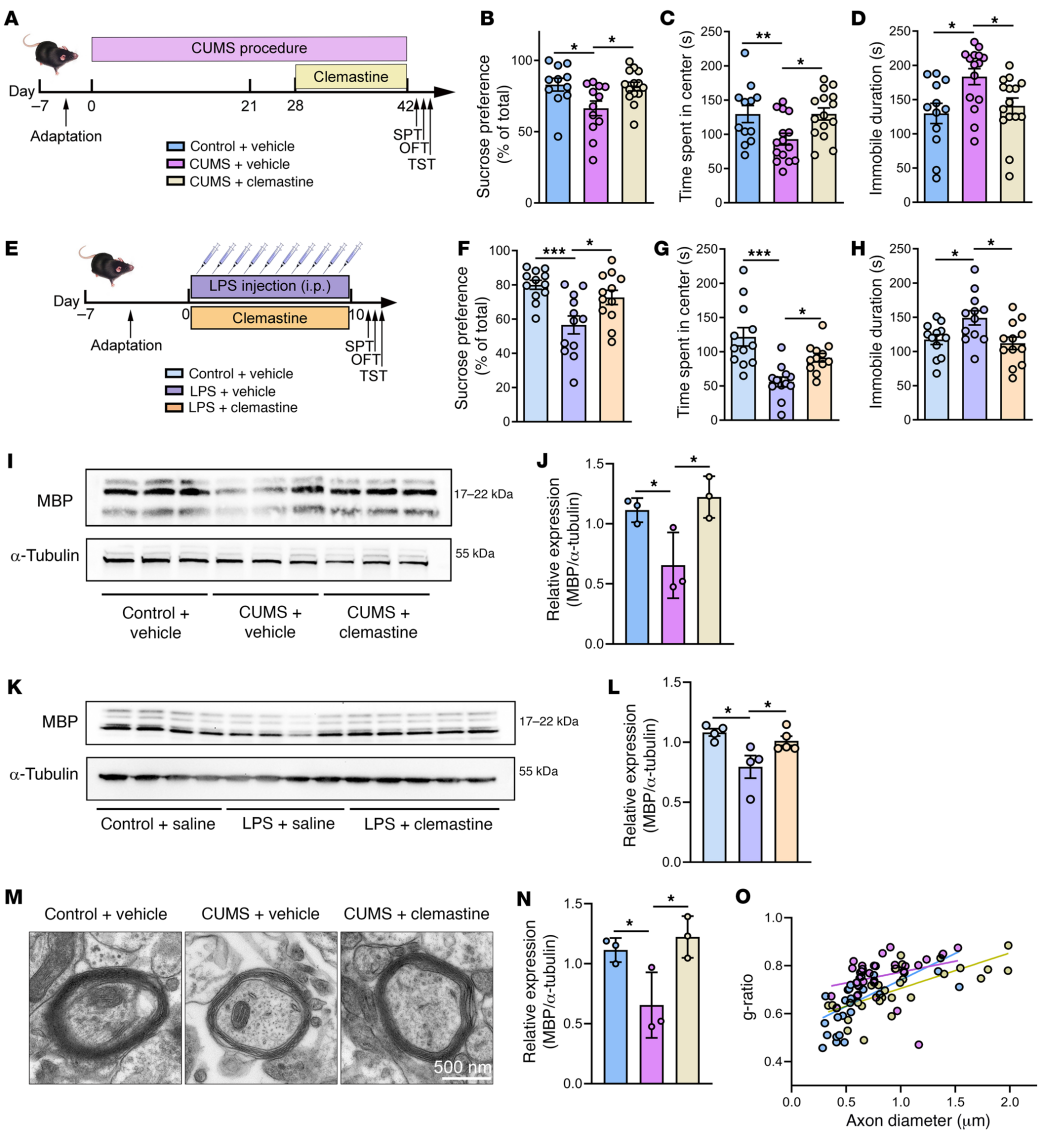
Clemastine promotes myelination and rescues depression-related behaviors in mice. (**A**) Schematic outline of clemastine treatment experiment in CUMS mice. (**B**–**D**) Behavioral testing of CUMS mice and clemastine treatment: (**B**) SPT [*F* (2, 34) = 4.657, CUMS plus vehicle: *n* = 12 mice; CUMS plus clemastine: *n* = 14 mice; control plus vehicle: *n* = 11 mice]; (**C**) OFT [*F* (2, 39) = 4.843]; and (**D**) TST [*F* (2, 39) = 5.197, CUMS plus vehicle: *n* = 15 mice; CUMS plus clemastine: *n* = 15 mice; control plus vehicle: *n* = 12 mice in the OFT and TST]. (**E**) Schematic outline of clemastine treatment experiment in LPS-treated mice. (**F**–**H**) Behavioral testing of LPS-treated mice and clemastine treatment: (**F**) SPT [*F* (2, 33) = 8.388]; (**G**) OFT [*F* (2, 33) = 12.13]; and (**H**) TST [*F* (2, 33) = 5.023] (*n* = 12 mice/group). (**I** and **J**) Western blots and analysis showing lower levels of MBP that were restored by clemastine treatment in CUMS mice [*n* = 3 brains/group, *F* (2, 6) = 7.113]. (**K** and **L**) Western blots and analysis showing that clemastine treatment restored the diminished expression of MBP caused by LPS [*n* = 4–5 brains/group, *F* (2, 10) = 6.098]. (**M**) Representative electron microscopic images of ventral hippocampus myelinated axons from CUMS mice treated with clemastine and from control groups (*n* = 8 images from 3 mice/group). Scale bar: 500 nm. (**N**) Clemastine restored decreased myelin sheath thickness in CUMS mice, based on measurements from electron microscopic images [*F* (2, 85) = 18.81]. (**O**) The g-ratio of the inner to outer diameter of myelin sheaths plotted against the axon diameter. Data are shown as the mean ± SEM. *P < 0.05, **P < 0.01, and ***P < 0.001, by 1-way ANOVA with Dunnett’s post hoc comparison.

To determine whether demyelination is the cause or consequence of depression-related behaviors in mouse models, we investigated whether remyelination would restore normal behavior in tests relevant to depression. Clemastine has remyelinating effects in animal models of MS and patients with MS (38). Thus, if demyelination is necessary for abnormal behavior in CUMS mice, pharmacological remyelination by clemastine should normalize these depression-related behaviors. We injected 10 mg/kg clemastine intraperitoneally each day into CUMS mice from days 29 to 42 and tested their behaviors from day 43 onward. As shown in Figure 3, A–H, clemastine rescued the behavioral deficits in both CUMS (Figure 3, A–D) and LPS (Figure 3, E–H) mice, normalizing the SPTs, OFTs, and TSTs. We then confirmed the remyelinating effect of clemastine by measuring MBP expression, which was restored to the same level as that in control animals in both the CUMS (Figure 3, I and J) and LPS (Figure 3, K and L) models. Electron microscopic analysis of the hippocampus revealed similar results at the ultrastructural level, showing that clemastine restored the myelin sheath to a normal thickness after being substantially thinned by CUMS (Figure 3, M and N). Quantification of myelin thickness relative to the axonal diameter (g-ratio) revealed that CUMS resulted in thinner myelin in mice treated with vehicle (g = 0.654 ± 0.019) compared with control mice treated with vehicle (g = 0.829 ± 0.056, *P* < 0.01). This was reversed when the CUMS mice were treated with clemastine (g = 0.701 ± 0.017, *P* < 0.05) (Figure 3O).

### Clemastine restores synaptic deficits.

**Figure 4:**
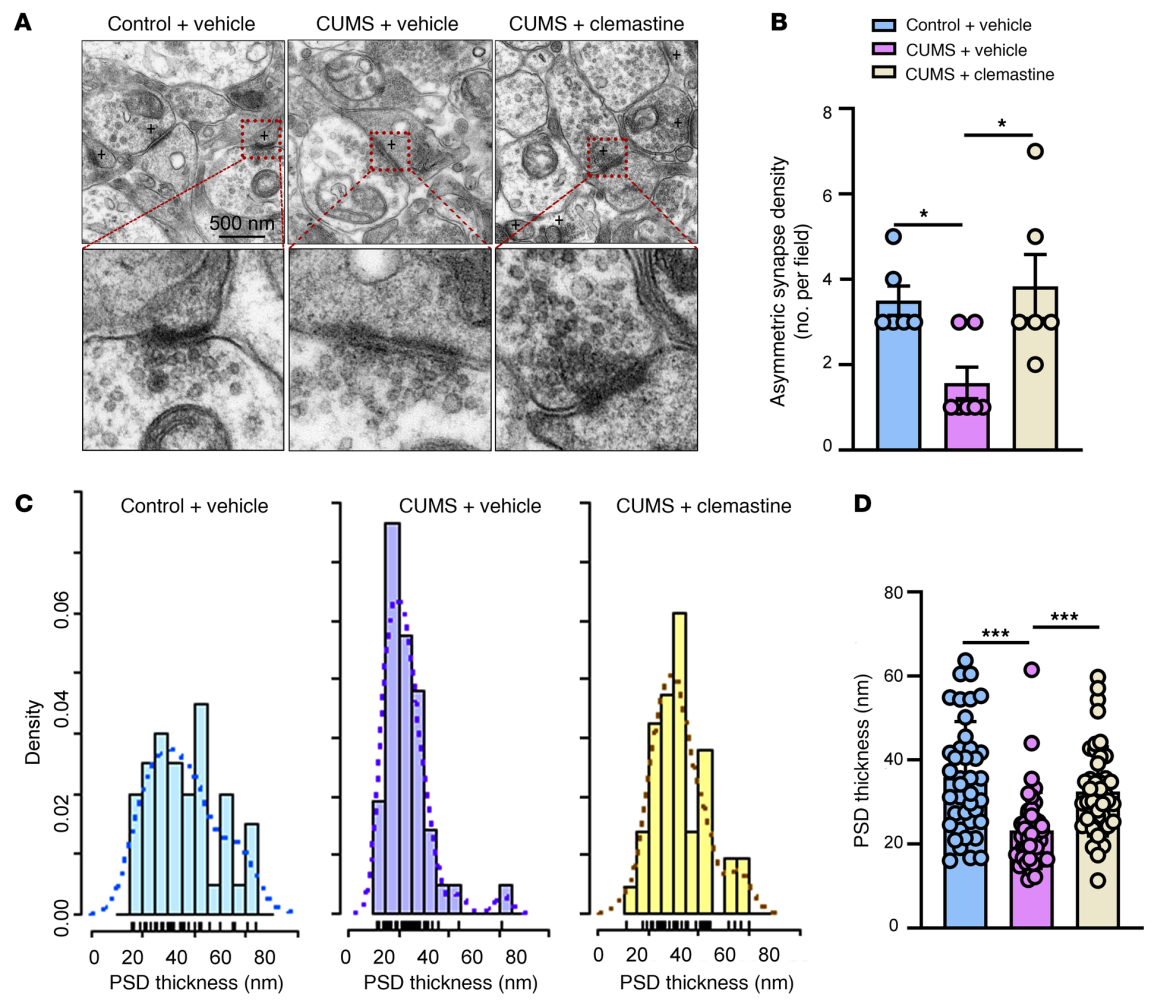
Clemastine reverses synaptic deficits. (**A**) Representative electron microscopic images of CUMS mice showing synaptic deficits that were rescued by clemastine (*n* = 3 mice/group). Scale bar: 500 nm. (**B**) The reduction in asymmetric synapses resulting from CUMS was rescued by clemastine [*F* (2, 16) = 6.063]. (**C**) Frequency distributions of PSD thickness. (**D**) Clemastine treatment normalized PSD thickness in CUMS mice to control levels [*n* = 40 asymmetric synapses from 3 mice in control and vehicle-treated groups; *n* = 42 asymmetric synapses from 3 mice in CUMS plus the vehicle group; *n* = 43 asymmetric synapses from 3 mice in the CUMS plus the clemastine-treated group, *F* (2, 122) = 14.50]. Data are shown as the mean ± SEM. *P < 0.05 and ***P < 0.001, by 1-way ANOVA with Dunnett’s post hoc comparison test.

We used electron microscopy to examine the synaptic ultrastructure in CUMS mice, because we hypothesized that there would be synaptic changes in addition to abnormal behaviors and demyelination. As shown in Figure 4, A and B, the number of asymmetric synapses (1.57 ± 0.37/field, *P* < 0.05) in CUMS mice was significantly reduced compared with that in control mice (3.50 ± 0.34/field), a deficit that was reversed by clemastine treatment (3.83 ± 0.75/field, *P* < 0.05). Moreover, the postsynaptic density in CUMS mice was thinner (35.95 ± 2.09 nm) than that in control mice (23.26 ± 1.40 nm, *P* < 0.001), and clemastine restored the postsynaptic density thickness to control levels (32.53 ± 1.62 nm, *P* < 0.001) (Figure 4, C and D). These data demonstrate that environmental stress can induce synaptic deficits that can be rescued by clemastine.

### Increased EphA4 receptor expression is associated with demyelination in CUMS mice.

**Figure 5:**
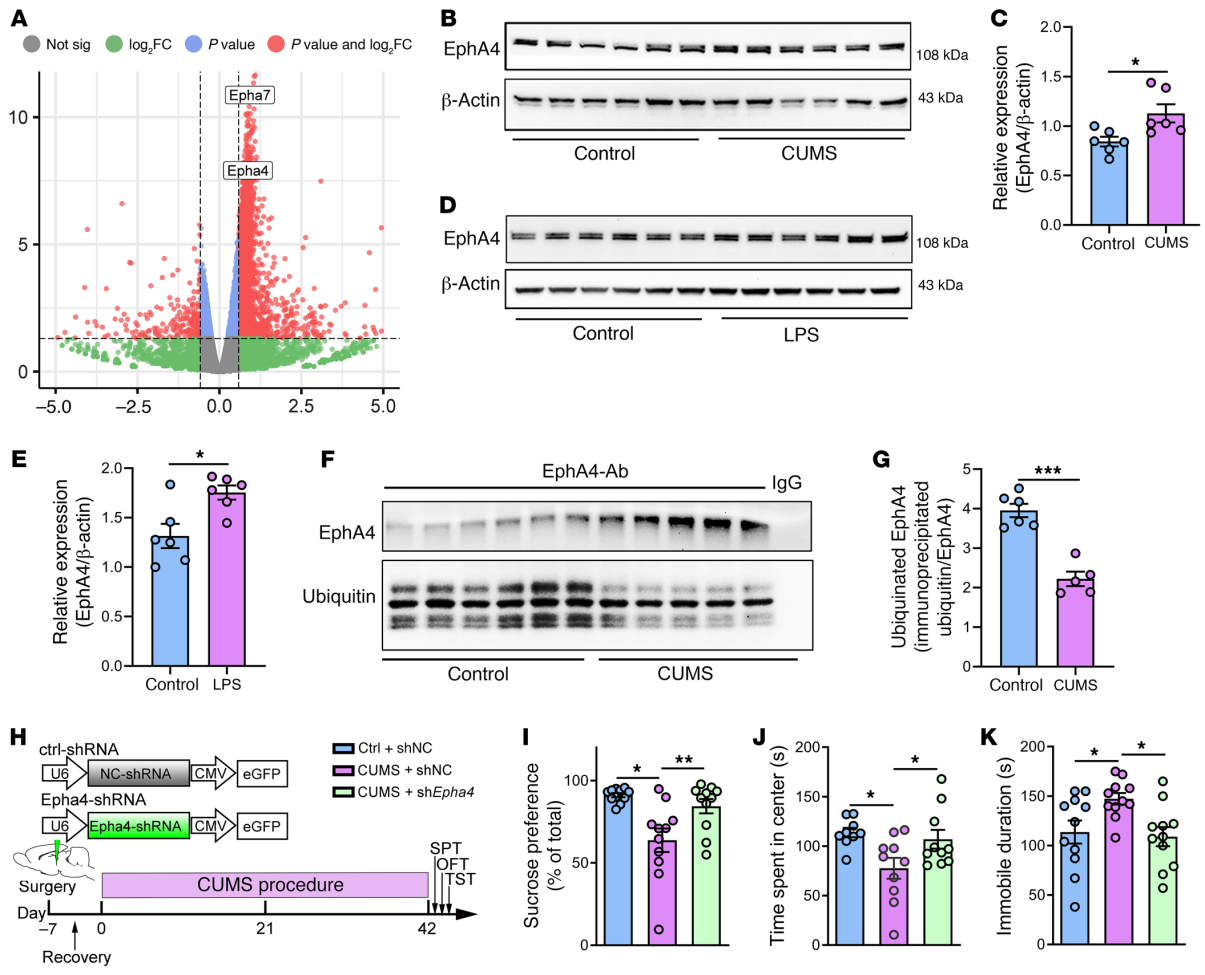
EphA4 knockdown rescues CUMS-induced depression-related phenotypes in mice. (**A**) Volcano plot of DEGs in CUMS mice versus controls. Cutoff values for the adjusted *P* value and fold change were set at 0.05 and 1.5, respectively. (**B** and **C**) Western blot and analysis showing increased EphA4 in hippocampus after CUMS (*n* = 6 mice/group, *t*_10_ = 2.756). (**D** and **E**) Western blot and analysis showing increased EphA4 in hippocampus after LPS injection (*n* = 6 mice/group, *t*_10_ = 3.080). (**F** and **G**) The level of ubiquitinated EphA4 was dramatically decreased in CUMS mice (*n* = 5–6 mice/group, *t*_9_ = 6.918). (**H**) Diagram outlining the layout of the AAV shRNA vector used to knock down EphA4 and the experimental timeline. (**I** and **K**) Behavioral effects of EphA4 knockdown in the (**I**) SPT [*F* (2, 30) = 8.580]; (**J**) OFT [*F* (2, 26) = 4.712; and (**K**) TST [*F* (2, 30) = 4.961] in CUMS mice (*n* = 9–11 mice/group). Data are shown as the mean ± SEM. *P < 0.05, **P < 0.01, and ***P < 0.001, by 1-way ANOVA with Dunnett’s post hoc comparisons test (**I**–**K**) and unpaired Student’s *t* test (**C**, **E**, and **G**).

In an effort to identify specific genes that might be responsible for demyelination in CUMS mice, we performed RNA-Seq on hippocampal tissue extracted from mice on day 21 and day 42 of the CUMS protocol. A volcano plot of differential gene expression in CUMS mice versus control mice was generated using the Bioinformatics tool (http://www.bioinformatics.com.cn), a free online platform for data analysis and visualization. We found 2 members from the Eph family of RTKs, *Epha4* and *Epha7*, that were among the top 100 upregulated genes (Figure 5A). The changes in mRNA levels of *Mbp*, *Dlg4*, and other genes involved in myelination such as *Sox10* and *Myrf* (39, 40) and synaptic genes such as *SAP97* (41) are shown in Supplemental Figure 4, A–L. Gene ontology (GO) analysis of the differentially expressed genes (DEGs) is shown in Supplemental Figure 4M.

We focused on EphA4, as the Eph family of RTKs have been implicated in the regulation of synapse development and plasticity (30). EphA4 is mainly expressed in the adult hippocampus as a suppressor of neurotransmission and synaptic plasticity (42). Increased EphA4 has been consistently observed after traumatic brain injury in humans (43). Similarly, EphA4, along with several other Eh receptors, appears to inhibit neuronal regrowth and recovery after spinal cord injury in mice (44). Increased expression of EphA4 has been observed in axonal lesions in MS, a demyelinating neurological disorder (45). Consistent with the RNA-Seq results, we confirmed by Western blotting that EphA4 was significantly increased in the hippocampus of CUMS mice (Figure 5, B and C) and LPS mice (Figure 5, D and E).

### Increased EphA4 receptor expression in CUMS might be a result of decreased ubiquitination.

To further investigate what induced the increase in EphA4 expression, we carried out a modified mass spectrometric analysis of proteins immunoprecipitated by an anti-EphA4 antibody in mouse brain. We found 463 proteins immunoprecipitated by the anti-EphA4 antibody, but not by the IgG control, in both control and CUMS mouse hippocampus. Further analysis of the 463 proteins showed that there are a marked number of proteins in the ubiquitin-mediated degradation pathway (Supplemental Table 1). Thus, we hypothesized that the observed enhancement of EphA4 expression may have been due to decreased ubiquitination in CUMS mice. To investigate this possibility, we first measured the level of ubiquitination in proteins extracted from CUMS mouse brain compared with levels in controls. As shown in Supplemental Figure 5, the ubiquitination of proteins was significantly decreased in CUMS (Supplemental Figure 5, A and B) and LPS (Supplemental Figure 5, C and D) mouse brains. We then measured the level of ubiquitinated EphA4 in CUMS mice using anti-EphA4 antibody to precipitate EphA4 followed by Western blot analysis with anti-ubiquitin antibody. As shown in Figure 5, F and G, the level of ubiquitinated EphA4 was dramatically decreased in CUMS mice, despite the significant upregulation in the directly immunoprecipitated EphA4, consistent with the increase in the expression of EphA4. These data suggest that the EphA4 upregulation induced by CUMS might be due to decreased ubiquitination.

### Eph A4 knockdown rescues the depression-related phenotypes and synaptic deficits in mice caused by CUMS.

To investigate whether the association between increased EphA4 and CUMS was causal, we used a genetic knockdown strategy. We hypothesized that if EphA4 function is necessary for CUMS to produce demyelination and behavior changes, then reducing EphA4 expression should prevent the deficits caused by CUMS. We generated an adeno-associated viral (AAV) vector encoding EphA4 shRNA and a control shRNA AAV and injected these into mice before exposure to CUMS (Figure 5H). EphA4 shRNA knockdown was confirmed with Western blotting (Supplemental Figure 6).

**Figure 6:**
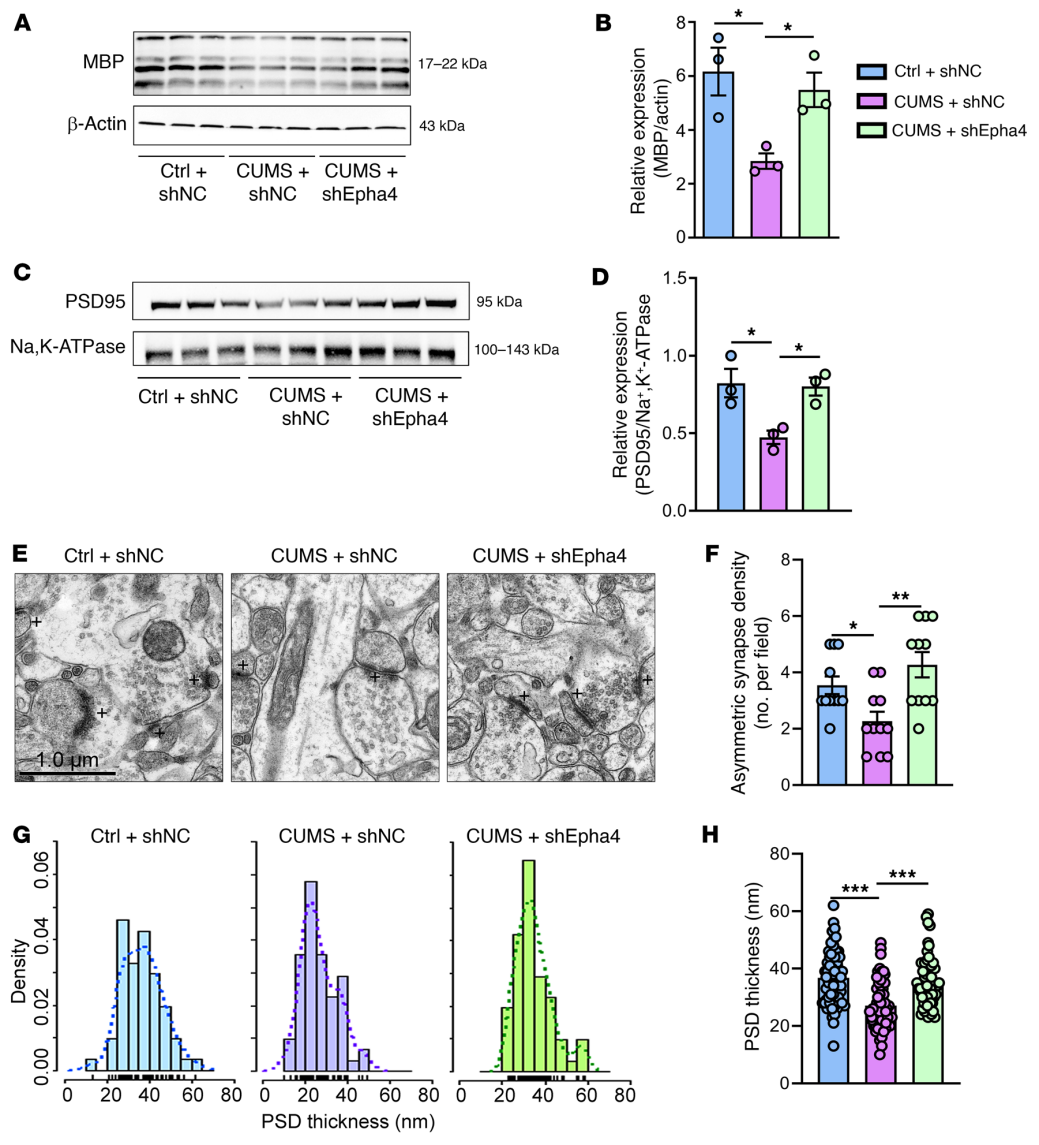
EphA4 knockdown in mice rescues synaptic deficits caused by CUMS. (**A** and **B**) Western blot analysis showing lower levels of MBP in CUMS mice restored by EphA4 knockdown [*n* = 3 brains/group, *F* (2, 6) = 7.264]. (**C**) Representative Western blot images of PSD95 protein levels; Na^+^K^+^ATPase was used as the protein loading control. (**D**) Densitometric analysis of PSD95 levels shows that EphA4 knockdown restored the decrease caused by CUMS versus control levels [*n* = 3 brains/group, *F* (2, 6) = 8.407]. (**E**) Representative electron microscopic images of ultrastructure of synapses from the 3 treatment groups. Scale bar: 1.0 μm. (**F**) Quantification of asymmetric synapse density, showing that EphA4 rescued the decrease caused by CUMS [*n* = 11 images from 3 mice/group, *F* (2, 30) = 7.500]. (**G**) Histograms showing the differential distribution patterns of the PSD thickness. (**H**) EphA4 knockdown restored the reduced PSD thickness caused by CUMS [*n* = 61 asymmetric synapses analyzed from 3 mice in the control plus the shNC group, *n* = 62 asymmetric synapses from 3 mice in CUMS plus the shNC and CUMS plus shEpha4 groups, *F* (2, 182) = 21.79]. Data are shown are shown as the mean ± SEM. *P < 0.05, **P < 0.01, ***P < 0.001, by 1-way ANOVA with Dunnett’s post hoc comparison (**B**, **D**, **F**, and **H**). ctrl, control.

EphA4 knockdown prevented the depression-related behaviors in CUMS mice; sucrose preference (Figure 5I), open field behavior (Figure 5J), and TSTs (Figure 5K) were not significantly different than for the control mice. We obtained similar results with myelination, as demonstrated by measuring MBP levels (Figure 6, A and B), and with synaptic deficits, by quantifying PSD95 expression levels (Figure 6, C and D). Furthermore, EphA4 knockdown prevented the decrease in asymmetric synapses caused by CUMS, as seen by electron microscopy (Figure 6, E and F). Moreover, the distribution of PSD thickness in CUMS mice treated with shNC shifted toward a lower value than that in control mice treated with shNC, and this change in distribution pattern could be partially reversed by EphA4 knockdown (Figure 6G). Statistically, EphA4 knockdown significantly prevented thinning of the PSD by CUMS (Figure 6H).

### Enhanced expression of EphA4 in excitatory neurons is associated with demyelination in CUMS mice.

**Figure 7:**
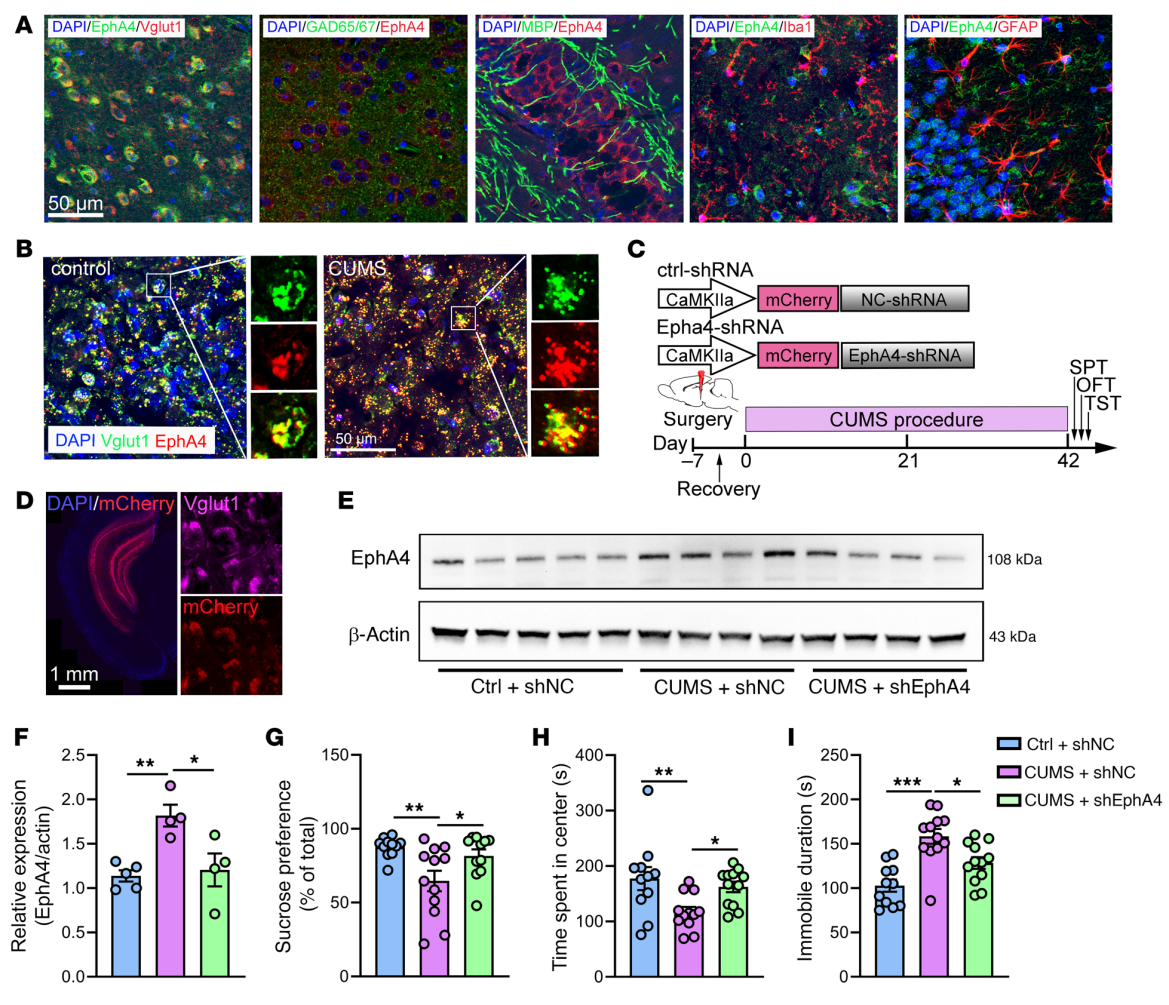
Specific knockdown EphA4 expression in excitatory neurons can rescue the depressive phenotypes in mice induced by CUMS. (**A**) EphA4 was mainly colabeled with Vglut1 in the ventral hippocampus of normal mice. (**B**) EphA4 expression in excitatory neurons was markedly increased by CUMS. Scale bars: 50 μm (*n* = 5 mice/group). (**C**) Diagram outlining the layout of the AAV shRNA vector used and the experimental timeline. (**D**) Representative images demonstrating that the AAV vectors can specifically infect excitatory neurons in the ventral hippocampus. Scale bar: 1 mm. (**E**) Knockdown efficiency of EphA4 shRNA vectors. (**F**) Increased levels of EphA4 in CUMS mice were restored by EphA4 shRNA treatment [*n* = 4–5 brains/group, *F* (2, 10) = 8.812 ]. (**G**–**I**) Behavioral effects of EphA4 knockdown in excitatory neurons in the (**G**) SPT [*F* (2, 31) = 5.814]; (**H**) OFT [*F* (2, 32) = 5.210]); and (**I**) TST [*F* (2, 32) = 14.18] in CUMS mice (*n* = 11–12 mice/group). *P < 0.05, **P < 0.01, ***P < 0.001.

We have shown that knockdown the expression of EphA4 in hippocampus prevented depressive-like behavior, demyelination, and synaptic deficits. Next, we sought to determine the specific cell type in which EphA4 is acting to produce these effects. Thus, we examined the expression of EphA4 using confocal microscopy with specific cell makers. As shown in Figure 7A, we found that in normal mouse brain, EphA4 was mainly coexpressed with Vglut1, a marker for excitatory neurons. In contrast, EphA4 was not colocalized with GAD65 and GAD67 (expressed in inhibitory neurons), MBP (expressed in oligodendrocytes), Iba1 (expressed in microglia), or GFAP (expressed in astrocytes), suggesting that EphA4 is mainly expressed in excitatory neurons. We then confirmed that the expression of EphA4 in excitatory neurons was substantially increased in CUMS mice (Figure 7B).

**Figure 8:**
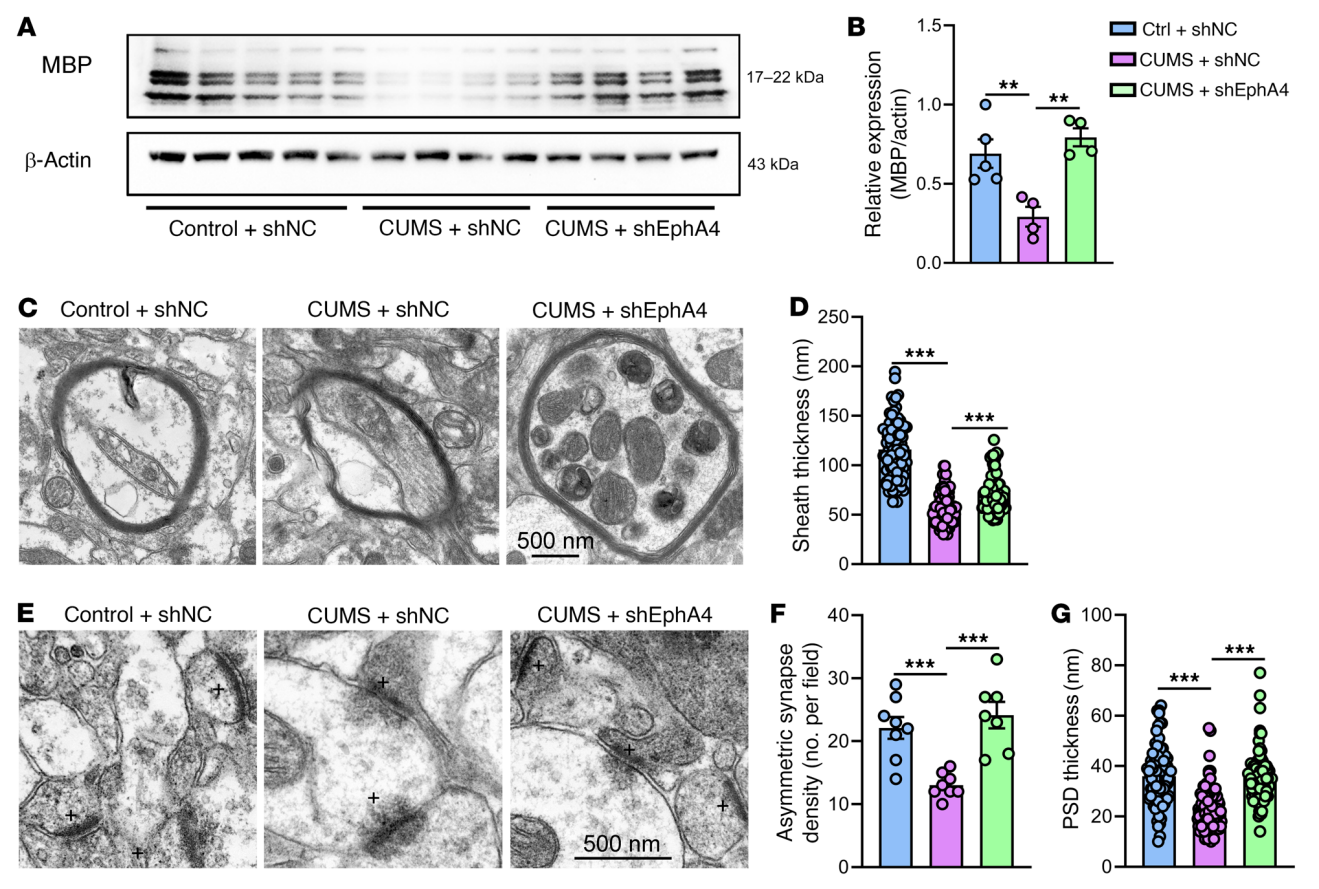
Specific knockdown of EphA4 expression in excitatory neurons in mice can inhibit demyelination and rescue the synaptic deficits induced by CUMS. (**A** and **B**) Lower levels of MBP in CUMS mice restored by EphA4 knockdown in excitatory neurons [*n* = 4–5 brains/group, *F* (2, 10) = 11.51]. (**C**) Representative electron microscopic images of myelinated axons. Scale bar: 500 nm. (**D**) Specific knockdown of EphA4 in excitatory neurons restored decreased myelin sheath thickness in CUMS mice [*n* = 85 myelinated axons from 3 mice/group, (*F* (2, 252) = 166.1]. (**E**) Representative electron microscopic images of synapses from 3 treatment groups. Scale bar: 500 nm. (**F** and **G**) EphA4 knockdown in excitatory neurons restored the reduced asymmetric synapse numbers (**F**) [*F* (2, 20) = 14.32] and PSD thickness (**G**) [*n* = 85 asymmetric synapses analyzed from 3 mice/group, *F* (2, 252) = 44.57] caused by CUMS. Data are shown as the mean ± SEM. **P < 0.01 and ***P < 0.001, by 1-way ANOVA with post hoc comparisons with Dunnett’s test.

To further confirm that EphA4 in excitatory neurons mediates demyelination in the pathogenesis of depression, we constructed an Epha4-knockdown vector with a CaMK2a promotor to specifically knock down the expression of Epha4 in excitatory neurons (Figure 7, C and D). Consistent with our prediction, EphA4 knockdown in excitatory neurons rescued the abnormal phenotype seen in WT CUMS mice, normalizing the increased expression of EphA4 (Figure 7, E and F), the depression-like behavior (Figure 7, G–I), the decreased expression of MBP (Figure 8, A and B), the decreased myelin sheaths (Figure 8, C and D), and the decreased number of asymmetric synapses (Figure 8, E and F) and thinning PSDs (Figure 8G).

### Altered levels of MBP, PSD95, and EphA4 in postmortem brain tissue from patients with MDD.

**Figure 9:**
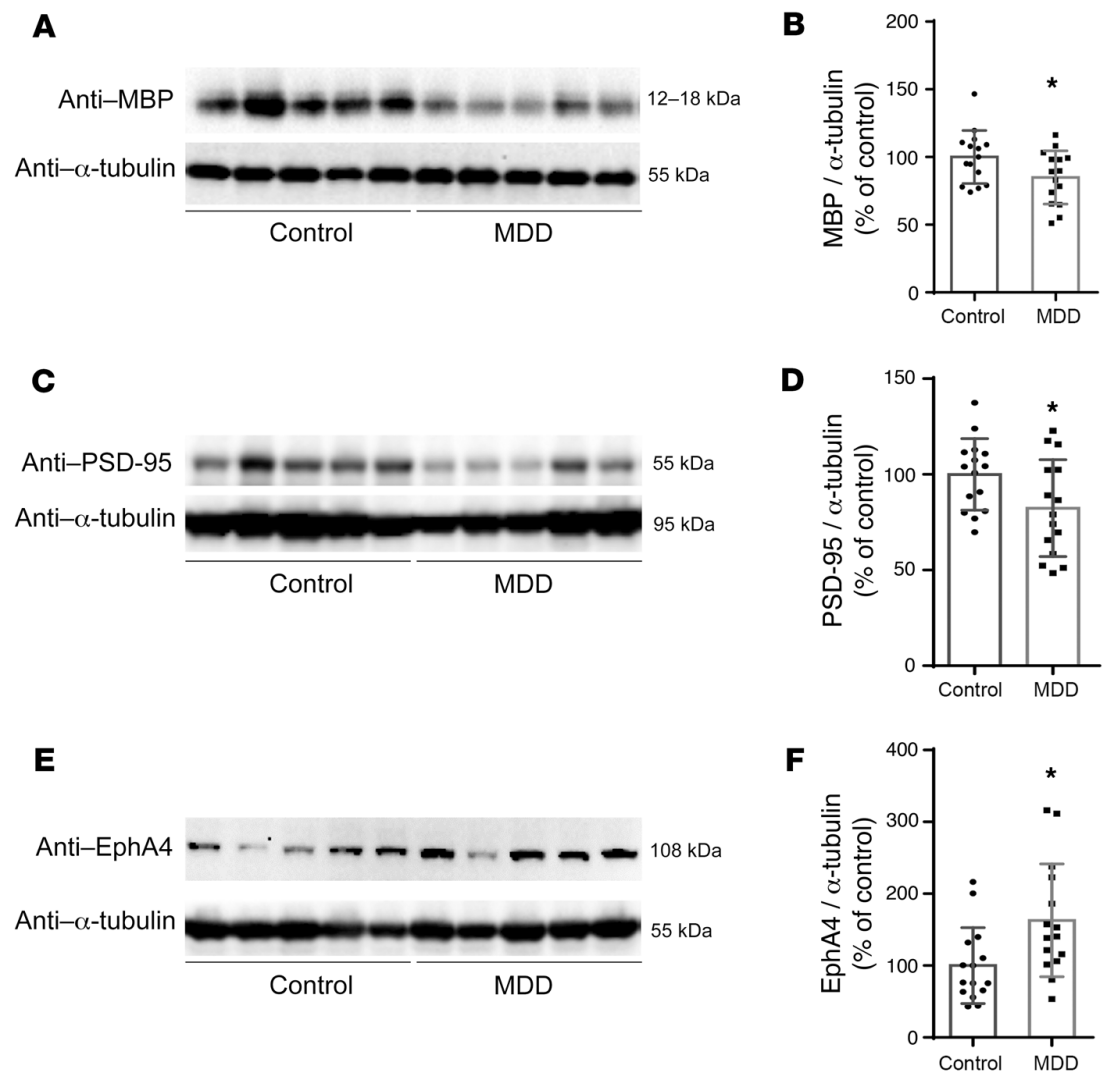
Altered levels of MBP, PSD95, and EphA4 in postmortem brain tissue from patients with MDD. (**A**) Representative Western blot images of MBP protein extracted from postmortem brain tissues donated by patients with MDD versus unaffected controls. (**B**) Densitometric analysis of MBP protein levels (*t*_28_ = 2.102). (**C**) Representative Western blot images of PSD95 protein extracted from postmortem brain tissues donated by patients with MDD versus unaffected control individuals. (**D**) Densitometric analysis of PSD95 protein levels (*t*_28_ = 2.171). (**E**) Representative Western blot images of EphA4 protein extracted from postmortem brain tissues donated by patients with MDD versus unaffected control individuals. (**F**) Densitometric analysis of EphA4 protein levels (*t*_28_ = 2.577). α-Tubulin was used as a loading control for all blots in this figure and *n* = 15 for all analyses. Data are shown as the mean ± SEM. *P < 0.05, by unpaired Student’s *t* test.

To translate our findings from mouse models of depression to humans, we measured the protein levels of MBP, PSD95, and EphA4 in postmortem brain samples provided by the Stanley Medical Research Institute (Rockville, Maryland, USA). We analyzed tissue from 15 patients with MDD and 15 unaffected control individuals, who were matched for age, sex, race, postmortem interval, pH (hydrogen ion concentration), side of brain, and mRNA quality (46, 47). The demographic information and antidepressant treatment data are shown in Supplemental Tables 2 and 3. Equal amounts of protein from each sample were immunoblotted with antibodies against either MBP, PSD95, or EphA4. Each Western blot included 5 samples from each group, and the results for each sample are presented as the percentage of the mean of 5 control samples on the same blot. Consistent with the results from CUMS mice, MBP and PSD95 protein levels were significantly decreased in patients with MDD compared with control individuals (*n* = 15, *P < 0.05; Figure 9, A–D), whereas EphA4 levels were significantly higher in MDD samples compared with levels in controls (*n* = 15, *P < 0.05; Figure 9, E and F).

## Discussion

We have demonstrated for the first time to our knowledge that increased EphA4 expression is necessary for physical or inflammatory stress to induce behaviors relevant to depression and demyelination in the mouse. Human data were consistent with the animal data, with increased EphA4 and decreased MBP levels in postmortem brain samples from patients with MDD. EphA4 knockdown prevented, and clemastine rescued, demyelination and behavior abnormalities in mouse models of depression. Thus, in our model systems, demyelination was necessary for environmental stress to cause a depression-like state, and remyelination was sufficient to restore normal function. We believe our work provides important insights about the neurobiology of depression. We also show the feasibility of inhibiting EphA4 specifically as a treatment strategy for depression and promoting remyelination as a more general approach to the development of new antidepressant medications.

It is well known that patients with MS often have depressive symptoms, but it has been unclear whether the depression is a nonspecific consequence of chronic, debilitating illness or if there is a direct pathophysiological link between the 2 disorders (48). Our data support previous associations between demyelination, oligodendrocyte dysfunction, and depression (25, 49, 50), with the additional discovery of a discrete molecular signal through EphA4 that is necessary for both the behavioral and cellular effects of the environmental stressors. Previous studies have reported more general evidence for glial cell involvement in depression, such as alterations in neurotransmitter levels and receptor expression in oligodendrocyte lineage cells in depression (51, 52). Clemastine has also been shown to be effective in promoting remyelination in MS, but not in the context of depression (38).

We also demonstrated that EphA4 was required to rescue the synaptic deficits caused by CUMS. There is previous evidence that enhancing myelination promotes synaptogenesis in the context of chronic hypoxia (53). Our data show that clemastine can significantly enhance synaptogenesis, which suggests that glial cells are important for synaptic integrity. Therefore, we believe our data are also significant, because they extend our knowledge of the functional role that myelination plays in regulating synaptogenesis in the context of depression.

Most neurobiological research on depression has focused on neurons, but more recent data suggest that MDD is also associated with glial pathology, which can affect the functioning of neural circuits in key brain regions related to depression (54–56). Both social and chronic stress can cause demyelination in animal models (7, 14, 15), which obviously implicates oligodendrocytes, since they are the main cell responsible for myelination (14, 22). Several recent studies provide direct evidence that focal induction of demyelination or focal ablation of oligodendrocyte precursor cells in specific brain areas is sufficient to induce depression-like behavior (24, 57). Complementary studies demonstrate that clemastine has antidepressant-like effects in mice (26). There is also loss of oligodendrocytes and myelin in cortical and subcortical areas in patients with depression and in mouse models of depression (14, 49, 57, 58).

The Eph family of RTKs have a diverse functional impact in the brain, including the regulation of neurogenesis, neuron migration, axon guidance, and synaptogenesis (59). The EphA4 receptor appears to suppress myelination in both the PNS and CNS (27, 28). This is consistent with the observation that increased EphA4 signaling in the mPFC modulates brain-derived neurotrophic factor (BDNF) signaling in the social defeat mouse model of depression (60). However, others have reported the seemingly contradictory observation of decreased EphA4 in the hippocampus of rats subjected to CUMS (61). This is also inconsistent with our data showing increased ventral hippocampal EphA4 expression after CUMS in mice, which would require further experiments to resolve. EphA4 binds a number of ligands, including ephexin 1 and ephrin A3, which modulate dendritic spine remodeling (60), and these may have different effects than those of oligodendrocyte-derived ligand interaction with EphA4. Nevertheless, there is further convergent evidence supporting our hypothesis. Cisplatin chemotherapy for cancer causes demyelination as a side effect (62),probably through activation of EphA4 by ROS-induced tyrosine phosphorylation (63).

Depression shows significant sex differences in prevalence and pattern of onset, such as that of postpartum depression (64). Demyelinating disorders such as MS also occur at higher rates in women (65). Because we only studied male mice, our data cannot automatically be generalized to female mice. It would be helpful in the future to collect data on female mice to determine whether or not the function of EphA4 in mediating both depression-like behavior and demyelination is similar in both sexes.

In summary, we have provided insights into the molecular mechanisms by which environmental stress is transduced into the cellular and behavioral abnormalities in depression. EphA4 appears to be the crucial hub that links disparate types of depression generating insults to demyelination, synaptic dysfunction, and behaviors relevant to depression. We believe our work illuminates the molecular explanation for the longstanding clinical observation that patients with MS also develop depression, and at the same time identifies EphA4 as a potential target for future antidepressant medication development. Future work could test whether the EphA4 inhibitor compound 1 (63) has antidepressant effects in mouse models.

## Methods

### Animals

Male C57BL/6J mice (7–8 weeks of age) were purchased from GemPharmatech. All animals were group housed on a 12-hour light/12-hour dark cycle with free access to food and water. Mice were allowed to habituate to this environment for at least 1 week before experiments were performed. Each cage housed a maximum of 4 mice.

### Animal models for studying depression and drug administration

#### CUMS procedure.

CUMS was performed as described previously (7, 35) with slight modifications. Briefly, male C57BL/6J mice were subjected to a continuous variety of mild stressors for 6 weeks, including food deprivation (24 hours), water deprivation (24 hours), cage tilt (45°, 7 hours), wet bedding (24 hours), light-dark reversal (24 hours), physical constraint (2 hours), forced swimming (4°C, 5 min), and tail pinch (1 min). These stressors were randomly scheduled for CUMS mice. Control mice were handled daily in the housing room. During the last 2 weeks of CUMS, mice in the treatment groups were given intraperitoneal injections of clemastine 10 mg/kg (Selleck Chemicals, S1847). The dose of clemastine was chosen on the basis of efficacy for increasing myelination according to previous studies (66).

#### LPS-induced depression-like state.

We used LPS to induce a depression-like state as previously described (7, 15). Male C57BL/6J mice (9–10 weeks of age) were given intraperitoneal injections of LPS (MilliporeSigma, L-2280) dissolved in sterile 0.9% saline at a dose of 0.5 mg/kg. Saline or LPS was injected between 09:30 am and 10:30 am for 10 consecutive days. Behavior tests were performed 24 hours after the last injection. In the clemastine-treated group, a dose of 10 mg/kg was injected intraperitoneally immediately after each LPS injection. The dose of LPS was chosen for its ability to induce depressive-like behaviors in mice without causing obvious inflammation (7).

### Behavior testing

#### SPT.

For the SPT, the mice were single housed and trained to consume 1% sucrose from 2 bottles. Mice were first habituated with 2 bottles of water for 24 hours, and then they were provided 2 bottles of 1% sucrose for another 24 hours. Mice were water deprived for 24 hours before the test. During the test, the mice had free access to either plain water or 1% sucrose from 2 different bottles, and the amount of each liquid consumed over 24 hours was recorded. Bottle positions were switched in the middle of the test. The sucrose preference index was defined as the weight of sucrose consumed, divided by the total weight of water and sucrose consumed.

#### OFT.

The OFT was used to observe locomotion and anxiety-like behavior in mice. Mice were transferred to the testing room and allowed to habituate for 2 hours prior to testing. Each mouse was initially placed in the center of the open field cage (40 × 40 × 30 cm) and allowed to freely explore for 15 minutes under dim light. Their behavior was recorded with a video tracking system positioned directly above the field, and the time spent in the center region (20 × 20 cm) was analyzed with Topscan (CleverSys).

#### TST.

Mice were individually suspended in the TST apparatus with paper adhesive tape placed approximately 1 cm from the tip of the tail. Their behavior was monitored using a video tracking system for 6 minutes. The test data were not analyzed if the mouse caught its tail with its paws. The immobility duration in the last 4 minutes was measured by 1 observer blinded to the treatment group’s identity. Immobility was defined as the amount of time the mouse hung passively or was motionless (67).

### Recombinant AAV2/9-shEpha4 plasmid construction

Recombinant AAV (rAAV) vectors (pscAV-U6-shRNA-CMV-GFP, constructed by Vigene Biosciences) carrying shRNAs against mouse *Epha4* and GFP or GFP alone were used to produce viruses. To construct the AAV vectors, the following sequence targeting the *Epha4* mouse gene was used: 5′-GGACTTGCAAGGAGACGTTTATTCAAGAGATAAACGTCTCCTTGCAAGTCCTTTTTT-3′. The titers of each virus were 1.09 × 10^13^ and 1.73 × 10^13^ viral genomes/mL, respectively, as measured by quantitative PCR (qPCR). For specific knockdown of *Epha4* in excitatory neurons, the rAAV vector (pAAV-CaMKIIa-mCherry-miR30shRNA-WPRE, constructed by Obio Technology) carrying shRNAs against mouse *Epha4* and mCherry or mCherry alone was used to produce virus. The titers of each virus were 6.41× 10^12^ and 1.58 × 10^13^ viral genomes/mL, respectively, as measured by qPCR.

### Stereotaxic surgery and virus injection

C57BL/6J mice were weighed and deeply anesthetized with intraperitoneal sodium pentobarbital (50 mg/kg) and were then mounted onto a stereotaxic frame (RWD Life Science Co., 68044). A volume of 0.5 μL virus was injected into the ventral hippocampus bilaterally using a laboratory syringe infusion pump via a 5 μL microsyringe (Hamilton) at a rate of 0.1 μL/min. The injection site coordinates were as follows: anteroposterior (AP), –3.16 mm; mediolateral (ML), ±2.90 mm; dorsoventral (DV), –4.00/–2.50 mm. After injection, the microsyringe was left for an additional 5 minutes before slowly being withdrawn.

### RNA-Seq analysis

The ventral hippocampus from control and CUMS-treated mice was dissected for RNA-Seq. Total RNA was extracted using TRIzol Reagent (Invitrogen, Thermo Fisher Scientific, 15596026) according to the manufacturer’s protocol. Construction of the cDNA library and sequencing were performed by Guangzhou RiboBio Company. Clean reads were obtained after discarding reads containing adapter, poly-N, or low-quality raw data. High-quality reads were aligned to the mouse reference genome mm10 with HISAT2. Next, HTseq was used to convert aligned short reads into read counts. DEGs were chosen according to the criteria of a fold change of greater than 1.5 and an adjusted *P* value of less than 0.05.

### Western blotting

The hippocampus was quickly dissected after the animals were anesthetized with isoflurane and euthanized. For total protein extraction, tissues from the dorsal and ventral hippocampus were homogenized in M-PER Mammalian Protein Extraction Reagent (Thermo Fisher Scientific, 78501) for 1 minute with a tissue homogenizer (Servicebio, KZ-II). Subsequently, supernatants were collected by centrifuging the brain homogenate at 13,200*g* for 10 minutes at 4°C. For membrane fraction extraction, the Membrane and Cytosol Protein Extraction Kit (Beyotime Biotechnology, P0033) was used according to the protocols provided. After protein extraction, the concentrations of each sample were determined by bicinchoninic acid (BCA) assay (Thermo Fisher Scientific, 23225). Finally, 15–20 μg protein samples were loaded and separated on 12%–15% SDS-PAGE gels. Antibodies against PSD95 (Servicebio, GB11277, 1:800); Na^+^/K^+^-ATPase (SAB, 48318, 1:5000); MBP (Servicebio, GB12226, 1:10,000); EphA4 (Abcam, ab5396, 1:1000); ubiquitin (MilliporeSigma, 07-2130, 1:2000); and α-tubulin (Bioworld Technology, BS1699, 1:5000) were used. ImageJ (NIH) was used to quantify band intensities. See complete, unedited blots in the supplemental material.

### Immunohistochemistry

Animals were anesthetized with sodium pentobarbital and perfused transcardially with ice-cold saline followed by 4% paraformaldehyde. Then, the brains were isolated and perfused in 30% sucrose in PBS. Coronal brain sections (25–30 μm) were cut by microtome (Leica, CM1860) and stored at 4°C. Brain slices were washed in PBS 3 times, and then permeabilization and blocking were performed in PBS containing 0.3% Triton X-100, 1% BSA, and 10% goat serum at room temperature for 1 hour. Slices were incubated with the primary antibodies overnight at 4°C and were washed 3 times the next day with PBS containing 0.1% Triton X-100, followed by incubation with fluorescence-conjugated secondary antibodies (Abcam, 1:1000) for 1 hour at 37°C. Finally, after counterstaining with DAPI, images were captured with an Olympus confocal microscope. The following primary antibodies were used in the immunofluorescence assay: anti-EphA4 (Abcam, ab5396, 1:50 or Santa Cruz Biotechnology, sc-365503, 1:50); anti-GFAP (Abcam, ab68428, 1:500); anti-Iba1 (Wako, 019-19741, 1:1000); anti-Vglut1 (Servicebio, GB11821, 1:500); anti-GAD65 and anti-GAD67 (Santa Cruz Biotechnology, sc-365180, 1:100); and anti-MBP (Servicebio, GB12226, 1:800).

### LFB staining of myelin

For LFB staining, formalin-fixed mouse brains were embedded in paraffin, and 4 μm coronal sections were cut and stained with LFB. Then, the sections were counterstained with Nissl. The mean densities of the LFB staining were quantified and averaged using ImageJ.

### Transmission electron microscopy

For transmission electron microscopy, the animals were perfused transcardially and sacrificed. Brain tissues were fixed in PBS containing 1.25% glutaraldehyde and 2% paraformaldehyde and postfixed with 1% OsO_4_ in phosphate buffer (PB) for 2 hours. Tissue blocks of less than 1 mm^3^ in size were dehydrated and embedded in resin blocks and then cut into 60–80 nm ultrathin sections for further staining with 2% uranium acetate. Images were captured with a transmission electron microscope (Hitachi, HT7700). The number of asymmetric synapses was counted manually by an investigator blinded to the sample identity. The inner and outer diameter of the myelin sheath and the thickness of the PSD were calculated with ImageJ. The g-ratios of myelinated fibers in hippocampus were calculated as the ratio of the inner diameter to the outer diameter of the myelin sheath.

### Identification of EphA4-interacting proteins by mass spectrometry combined with immunoprecipitation

To identify EphA4-interacting proteins, protein lysate (total protein amount: 600–700 μg) from ventral hippocampus of control and CUMS mice was incubated with EphA4 antibody (Santa Cruz Biotechnology, sc-365503, 1:50) or normal mouse IgG (Santa Cruz Biotechnology, sc-2025, 1:100) in a rotator at 4°C for 16–18 hours. The next day, 40 μL Protein A/G magnetic beads (Bimake, B23201) was added to each sample, and rotational incubation vHip was continued for an additional 2 hours at room temperature. The immunoprecipitated complex was collected, and the supernatant was discarded. Then, magnetic beads were washed 3 times with ice-cold washing buffer (0.3% Triton X-100 in PBS) to diminish the unspecific binding. The immunoprecipitated samples were sent to Shanghai Applied Protein Technology Co. Ltd. for mass spectrometric analysis. For identification of the interaction between EphA4 and ubiquitin, the samples immunoprecipitated by EphA4 were incubated with anti-ubiquitin (MilliporeSigma, 07-2130, 1:2000) and anti-EphA4 (Abcam, ab5396, 1:1000) antibodies.

### Protein extraction and Western blot analysis of human brain

Postmortem human brain tissue samples were obtained from the Stanley Medical Research Institute and consisted of samples from 15 patients with MDD and 15 unaffected control individuals matched for age, sex, race, postmortem interval, pH, side of brain, and mRNA quality. Brain tissue was placed in lysis buffer (50 mM Tris-Cl, 150 mM NaCl, 2 mM EDTA, 0.5% sodium deoxycholate, 1% NP-40, 1% Triton X-100, 0.1% SDS) and a protease inhibitor cocktail (MilliporeSigma; 1:100, pH 7.4) and homogenized on ice. After gentle shaking at 4°C for 1 hour, the samples were centrifuged at 10,000*g* for 10 minutes to obtain the supernatant as the protein extract. The concentration of protein samples was measured by BCA assay (Pierce, Thermo Fisher Scientific). The samples were diluted to equal concentrations and boiled at 90°C–100°C for 5–10 minutes in Laemmli buffer (Bio-Rad) supplemented with 5% β-mercaptoethanol. Equal amounts of protein were loaded onto the gel and subjected to SDS-PAGE. After the transfer of proteins onto nitrocellulose, the membranes were incubated with the following primary antibodies: anti-MBP (Cell Signaling Technology, mouse, catalog 83683S, 1:1000); anti–EphA4/SEK (Abcam, rabbit, catalog ab5396, 1:1000); anti-PSD95 (Abcam, rabbit, catalog ab238135, 1:2000); and anti–α-tubulin (MilliporeSigma, mouse, catalog T6074-200 UL, 1:10,000). After washes with 1 × TBS plus 0.05% Tween-20, the blots were incubated with the corresponding HRP-conjugated secondary antibodies and illuminated using ECL Plus reagents (Amersham). The blots were then imaged using ChemiDoc MP (Bio-Rad), and densitometric analysis of the bands was conducted using ImageLab (Bio-Rad).

### Statistics

Before statistical analysis, the distribution of the data was assessed for normality using the Shapiro-Wilk test. For normally distributed data, differences between groups were assessed with the Student’s 2-tailed *t* test or ANOVA with post hoc comparisons using Dunnett’s test. GraphPad Prism 8 (GraphPad Prism 6 for human data, GraphPad Software) was used, and *P* values of less than 0.05 were considered statistically significant. All data are presented as the mean ± SEM.

### Study approval

All animal procedures were approved by the IACUC of Shanghai JiaoTong University.

## Author contributions

YL conducted all the animal experiments with technical support from YC and JN. PS performed the experiments with human samples. TY was involved in critical discussions related to synaptic deficits. FL designed and supervised the project and wrote the manuscript with YL and AHCW. All authors reviewed and gave final approval of the manuscript.

## Supplementary Material

Supplemental data

## Version 1 03/10/2022

In-Press Preview

## Version 2 04/15/2022

Electronic publication
