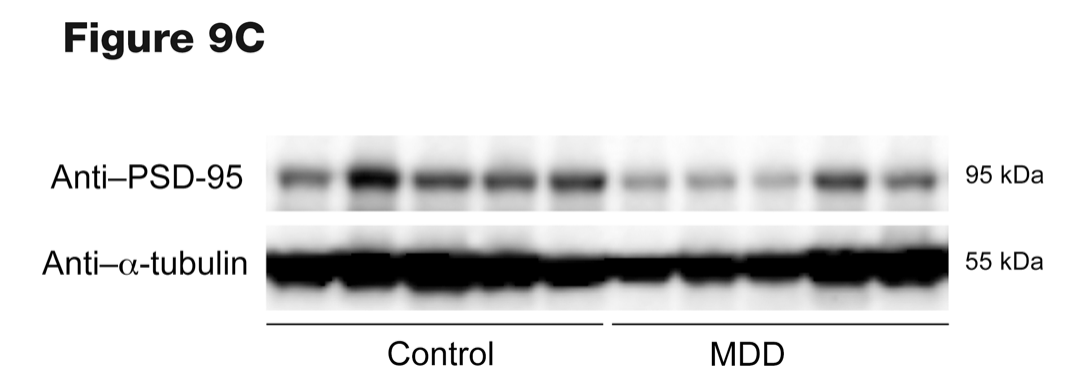# The Eph receptor A4 plays a role in demyelination and depression-related behavior

**DOI:** 10.1172/JCI161559

**Published:** 2022-05-16

**Authors:** Yuan Li, Ping Su, Yuxiang Chen, Jing Nie, Ti-Fei Yuan, Albert H.C. Wong, Fang Liu

Original citation: *J Clin Invest*. 2022;132(8):e152187. https://doi.org/10.1172/JCI152187

Citation for this corrigendum: *J Clin Invest*. 2022;132(10):e161559. https://doi.org/10.1172/JCI161559

Following the publication of this article, the authors became aware that an incorrect panel was used for Figure 3N. In addition, the molecular weight markers in Figure 9C were incorrect. The correct panels for Figures 3N and 9C are below. The article has also been corrected online.

The authors regret the error.

## Figures and Tables

**Figure:**
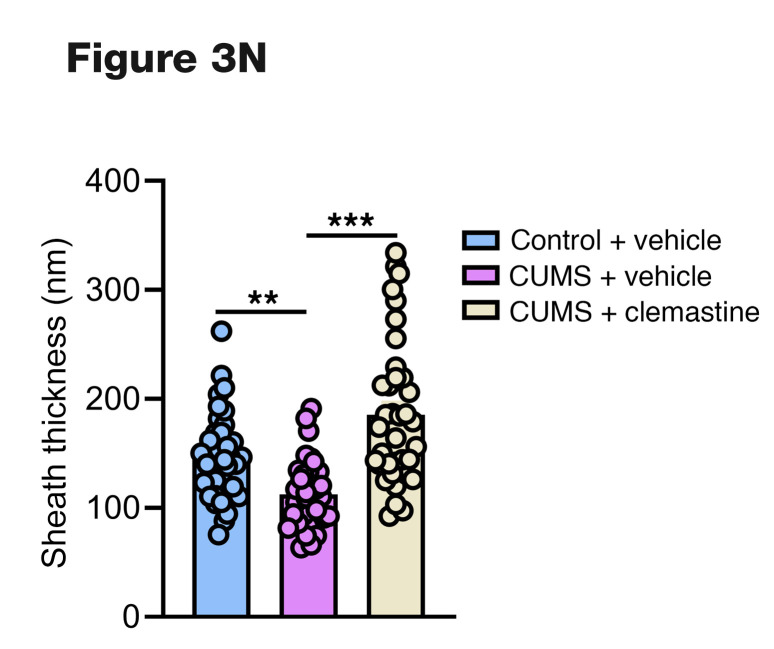


**Figure:**