# Supplementary material for: The Eph receptor A4 plays a role in demyelination and depression-related behavior
Source: J Clin Invest. 2022 Apr 15;132(8):e152187. doi: 10.1172/JCI152187 (PMC9012277; doi:10.1172/JCI152187)

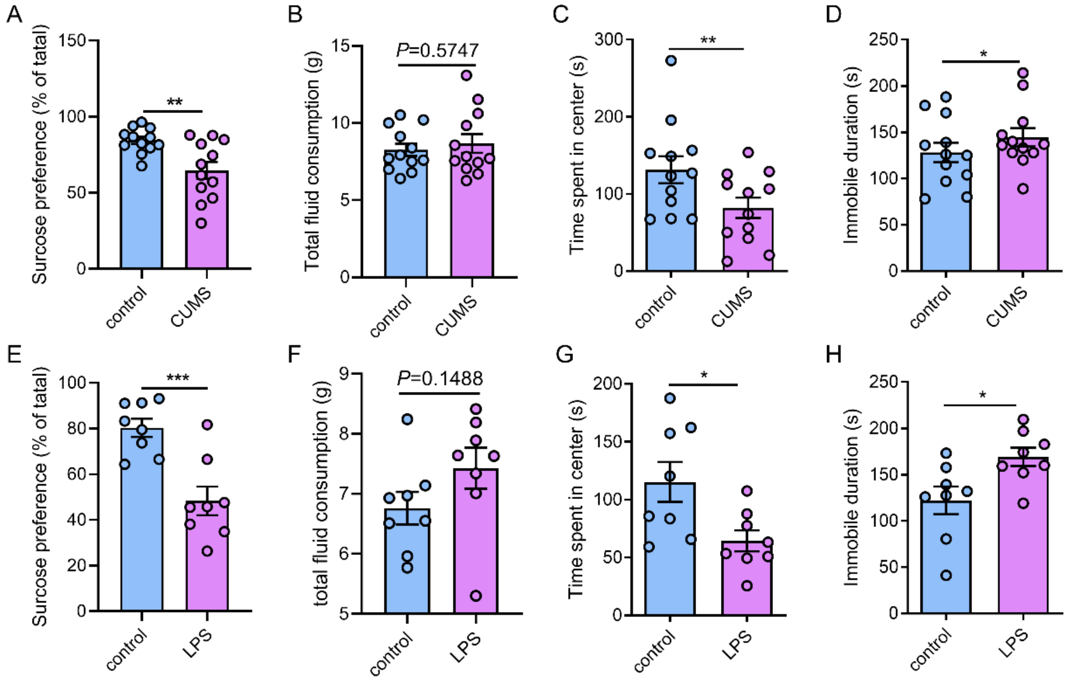

Supplemental Figure 1: Mice were exposure to chronic unpredictable mild stress (CUMS) or LPS to induce depressive-like behaviors. A-H: The effect of CUMS (**A-D**) and LPS (**E-H**) treatment on the behaviours of mice. The sucrose preference tests (SPT), open field test (OFT) and tail suspension test (TST) were used to evaluate the depressive-like state in mice (CUMS: n = 12/group; LPS: n = 8/group). The consumption of total fluid was also shown in **B** and **F**. Data are mean ± s.e.m. *$P<0.05$. Comparison between groups was performed by unpaired Student's t test.

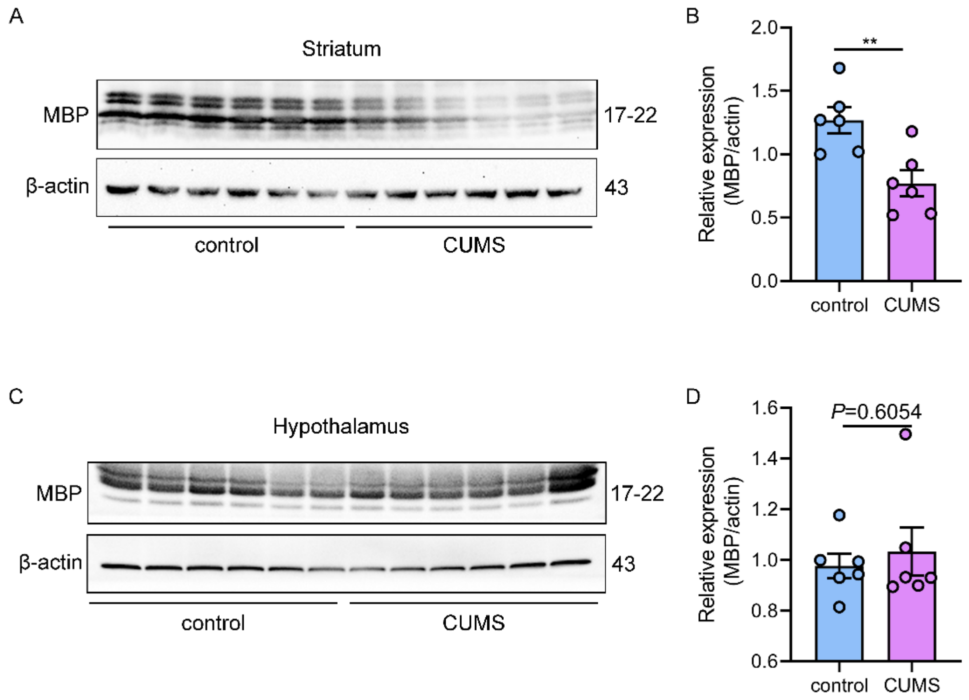

Supplemental Figure 2: Differential expression patterns of MBP protein in different brain regions from control and CUMS mice. A. Representative western blot images of MBP protein extracted from striatum in control and CUMS mice. **B.** Densitometric analysis of MBP protein levels (t_{10} = 3.438, n = 6 brains/group). **C.** Representative western blot images of MBP protein extracted from hypothalamus in control and CUMS mice. **D.** Densitometric analysis of MBP protein levels in C (t_{10} = 0.5334, n = 6 brains/group). Data are mean ± s.e.m. $^{**}P<0.01$. Comparison between groups was performed by unpaired Student's t test.

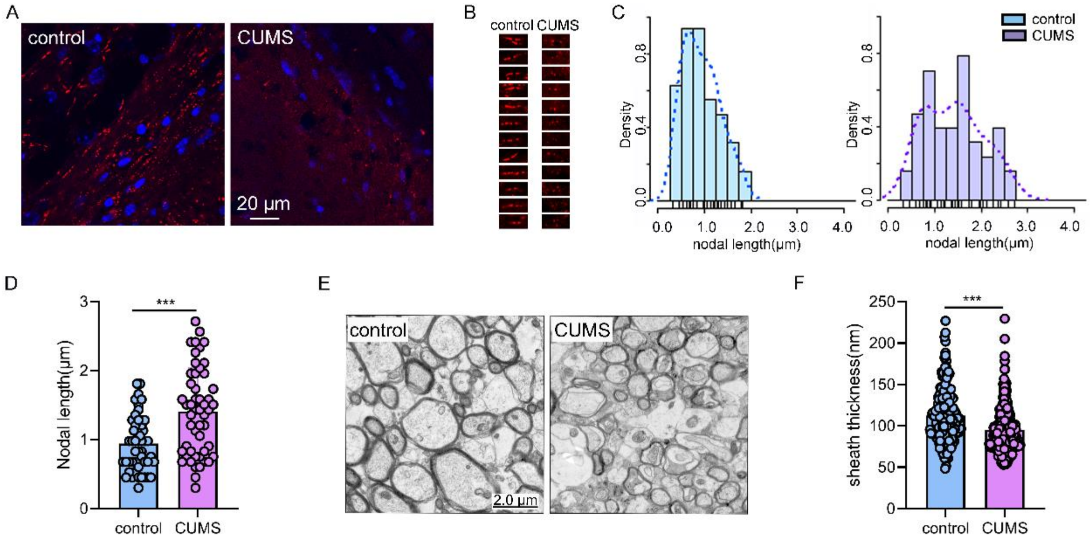

Supplemental Figure 3: Demyelination was observed in corpus callosum of CUMS mice. A. Representative images showing Caspr positive, red-stained paranodal regions in the corpus callosum. Scale bar: 20 µm (n = 4 mice/group). **B.** High magnification images of Caspr staining from A. **C-D.** Increased nodal length in the corpus callosum in CUMS mice, based on measurements of Caspr stained regions (n = 51 nodes from 3 different mice/group) (t_{100} = 4.489). **E.** Rrepresentative electron microscopy images showing demyelination in the corpus callosum in CUMS mice. Scale bar: 2.0 µm. **G.** Thinner myelin sheath in CUMS mice, measured from electron microscopic images (The number of myelin sheath analyzed from 3 mice/group was 210 and 270, respectively, t_{478} = 6.455). Data are mean ± s.e.m. *** $P< 0.001$. Comparison between groups was performed by unpaired Student’s t test.

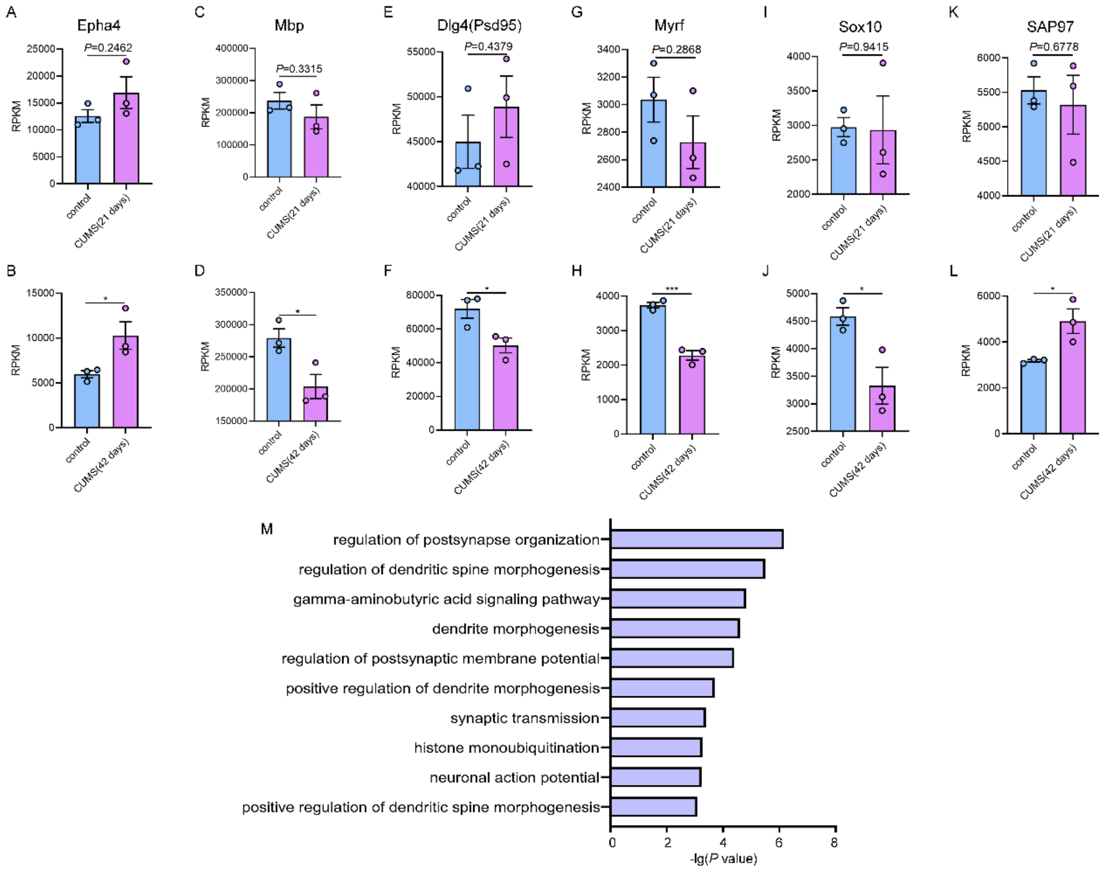

Supplemental Figure 4: The temporal mRNA dynamics of genes related to myelination and synapse in response to the CUMS intervention. The mRNA expression levels of Epha4 (**A-B**), Mbp (**C-D**), Psd95 (**E-F**), Myrf (**G-H**) Sox10 (**I-J**) and SAP97 (**K-L**) were shown. **M.** GO terms corresponding to differentially expressed genes in the hippocampus. Data are mean ± s.e.m. * *P*< 0.01. Comparison between groups was performed by unpaired Student’s t test.

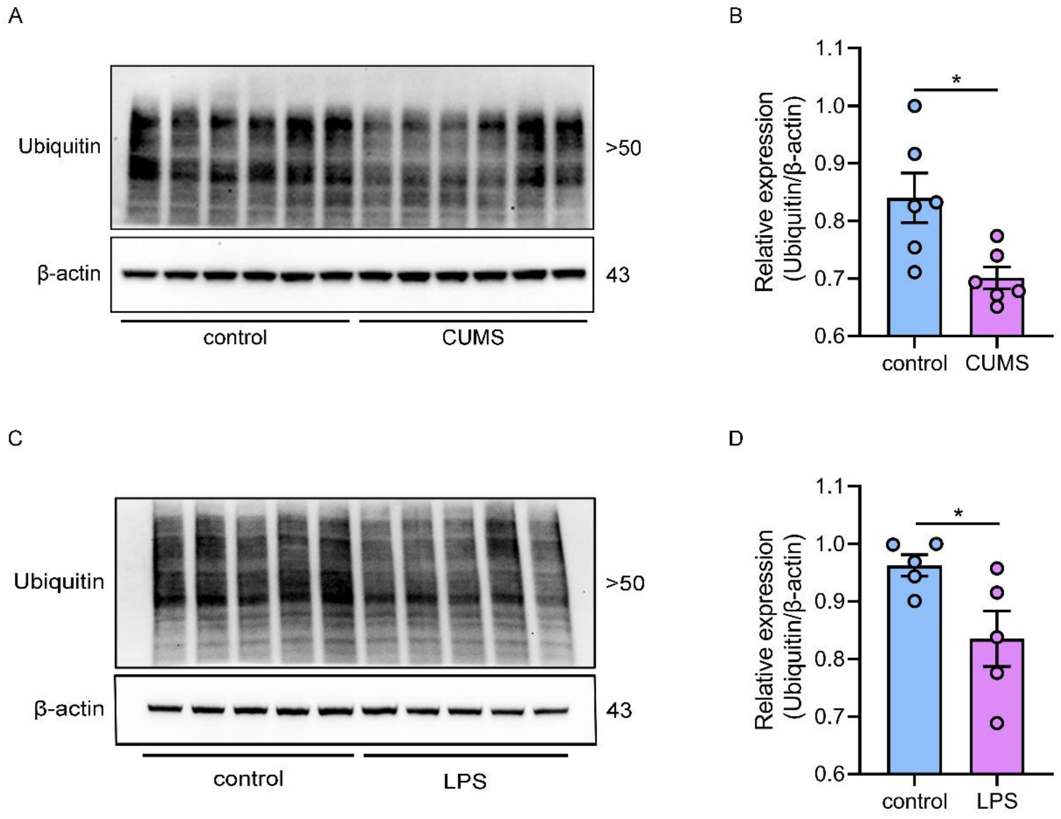

Supplemental Figure 5: Decreased level of ubiquitinated proteins in brain samples from CUMS mice and LPS mice. A. Representative western blot images of ubiquitinated protein levels in CUMS mice. **B.** Densitometric analysis of ubiquitinated protein levels protein levels ($t_{10} = 2.944$, n = 6 brains/group). **C.** Representative western blot images of ubiquitinated protein levels in LPS mice. **D.** Densitometric analysis of ubiquitinated protein levels in C ($t_8 = 2.469$, n = 5 brains/group). Data are mean ± s.e.m. * $P < 0.01$. Comparison between groups was performed by unpaired Student's t test.

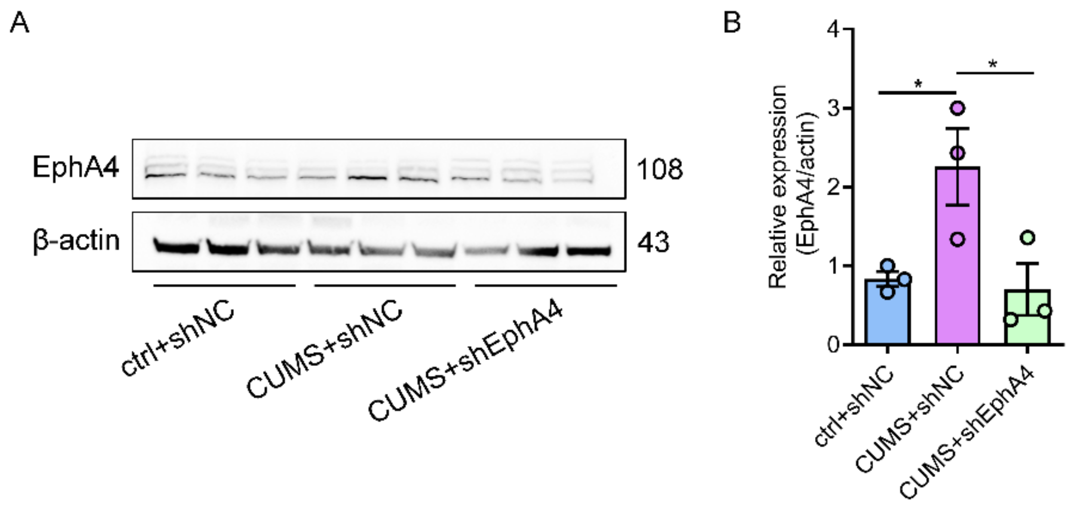

Supplemental Figure 6: CUMS-induced upregulation of EphA4 can be significantly reduced by EphA4 knockdown. A. Representative Western blot images of EphA4 expression levels in control with shNC, CUMS with shNC and CUMS with shEpha4 mice. β-actin was used as loading control. **B.** Densitometric analysis of expression levels of EphA4 (n = 3/group, F (2, 6) = 6.266). Data are mean ± s.e.m. **P*<0.05. Comparison between groups was performed by one-way ANOVA with *post hoc* comparisons using Dunnett's test.

Accession	Description
A0A0J9YVB7	E3 ubiquitin-protein transferase MAEA
P61089	Ubiquitin-conjugating enzyme E2 N
A0A2R8W6R3	E3 ubiquitin-protein ligase RBX1
Q8BGG7	Ubiquitin-associated and SH3 domain-containing protein B
P52479	Ubiquitin carboxyl-terminal hydrolase 10
Q7TPH6	E3 ubiquitin-protein ligase MYCBP2
A0A0X1KG61	E3 ubiquitin-protein ligase CBL
Q4U2R1	E3 ubiquitin-protein ligase HERC2
Q58E42	Ubiquitin carboxyl-terminal hydrolase (Fragment)
A0A0N4SVF7	RING-type E3 ubiquitin transferase
A2A9P8	RING-type E3 ubiquitin transferase (Fragment)
D3YVU0	Ubiquitin carboxyl-terminal hydrolase 46
A6PWR8	Ubiquitin carboxyl-terminal hydrolase 43
Q8K4P8	E3 ubiquitin-protein ligase HECW1
Q3UMT4	Ubiquitinyl hydrolase 1 (Fragment)
E9Q6Y8	Ubiquitin-specific peptidase 31
A0A087WRV6	E3 ubiquitin-protein ligase TRIP12 (Fragment)
F6RXM1	HECT and RLD domain-containing E3 ubiquitin protein ligase family member 1 (Fragment)
A0A338P6W1	E3 ubiquitin-protein ligase RBBP6 (Fragment)
A0A0R4J260	Ubiquitinyl hydrolase 1

Supplemental Table 1: List of EphA4-immunoprecipated proteins that are associated with ubiquitin signaling from control and CUMS-treated brain samples.

Variables	Control (N = 15)	MDD (N = 15)
Age (years) *	48 ± 11	47 ± 9
Sex	6 Female + 9 Male	6 Female + 9 Male
Onset of disease (age)	N/A	34±13
Disease duration (yrs)	N/A	13±11
pH	6.3±0.2	6.2±0.2
PMI (h)	24±10	27±11
Psychosis present (n)	N/A	0
Suicide (n)	N/A	7
History of Substance Abuse (n)	3	5
Severity of Substance Abuse	2 lowest<br>1 low	1 lowest<br>1 lower<br>1 higher<br>2 highest
History of Alcohol Abuse (n)	15	14
Severity of Alcohol Abuse	5 lowest<br>6 lower<br>2 low<br>2 high	4 lowest<br>5 lower<br>1 high<br>1 higher<br>3 highest
Side (Striatum)	8 left<br>7 right	9 left<br>6 right

Supplemental Table 2: General information on the brain samples from MDD patients and unaffected controls from the Stanley Foundation.

Patient ID	Anti-depressant drug use
P-1	On amitriptyline. In past, nortriptyline.
P-2	On trazadone.
P-3	On fluoxetine.
P-4	Recent fluoxetine and trazadone.
P-5	Untreated for 6 years.
P-6	Past sertraline but not recent.
P-7	Recent fluoxetine.
P-8	On nefazadone.
P-9	Never treated.
P-10	Buspirone and imipramine.
P-11	Recent sertraline.
P-12	On buspirone and venlafaxine.
P-13	On nortriptyline and clomipramine.
P-14	Recent fluoxetine and amitriptyline.
P-15	On fluoxetine and nefazadone.

Supplemental Table 3: Anti-depressant drug use information for the post-mortem major depression patient brain samples.

Full unedited gels

1. Full unedited gel for Figure 1F:

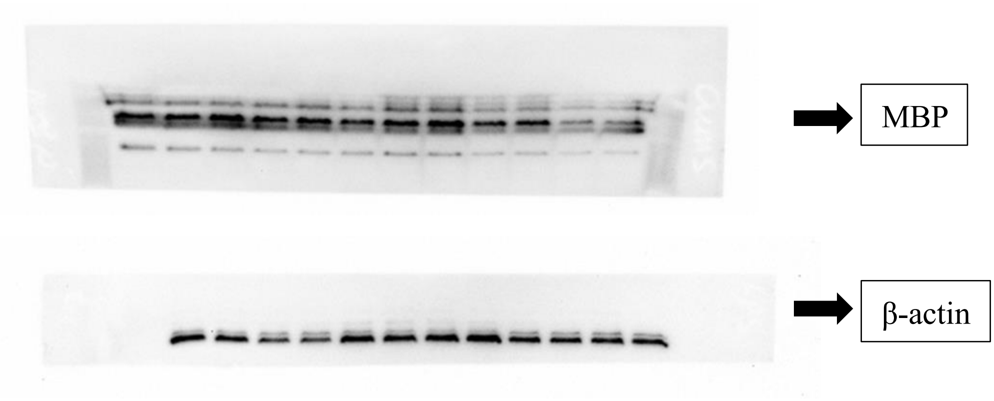

2. Full unedited gel for Figure 1F:

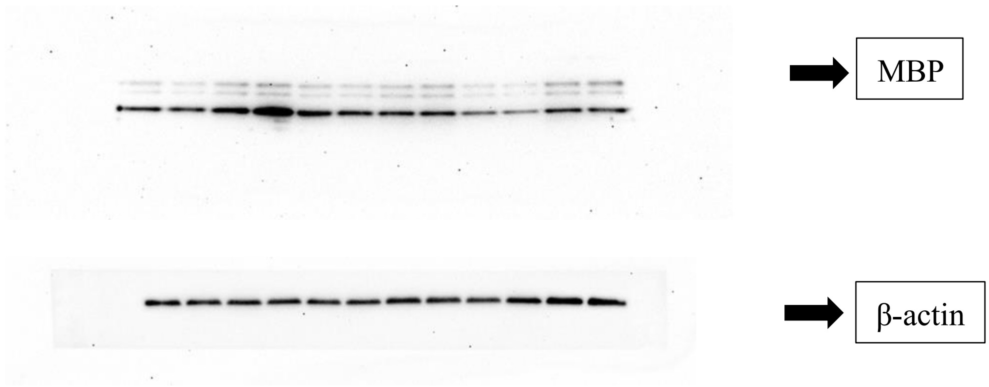

3. Full unedited gel for Figure 2H:

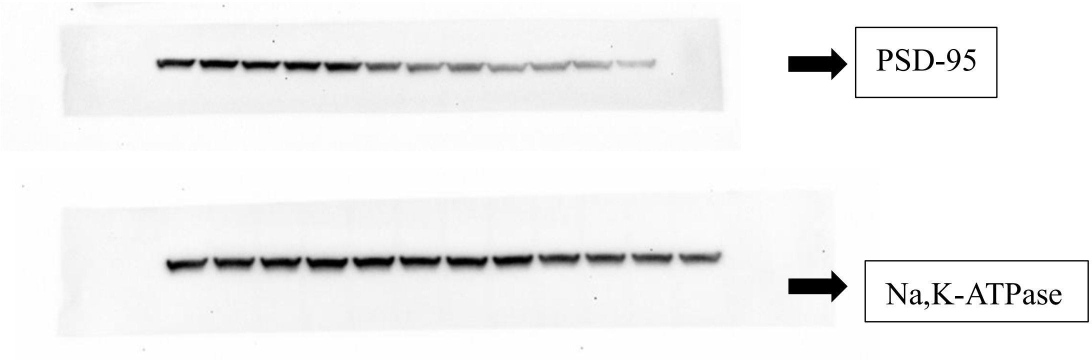

4. Full unedited gel for Figure 2J:

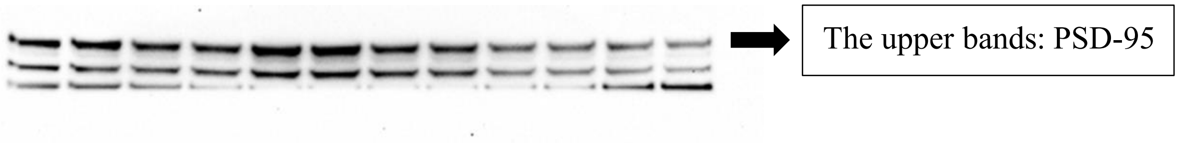

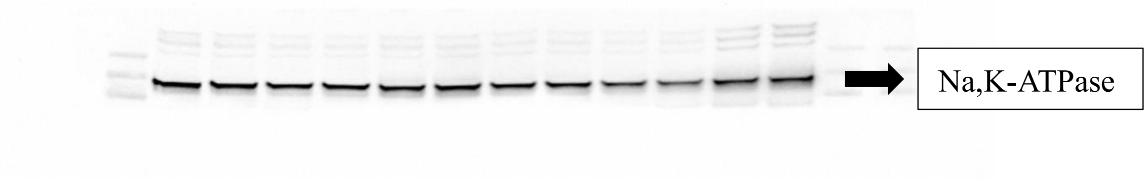

5. Full unedited gel for Figure 3I:

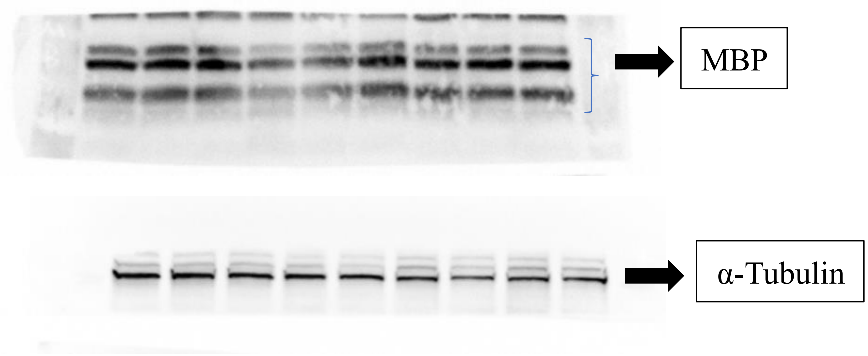

6. Full unedited gel for Figure 3K:

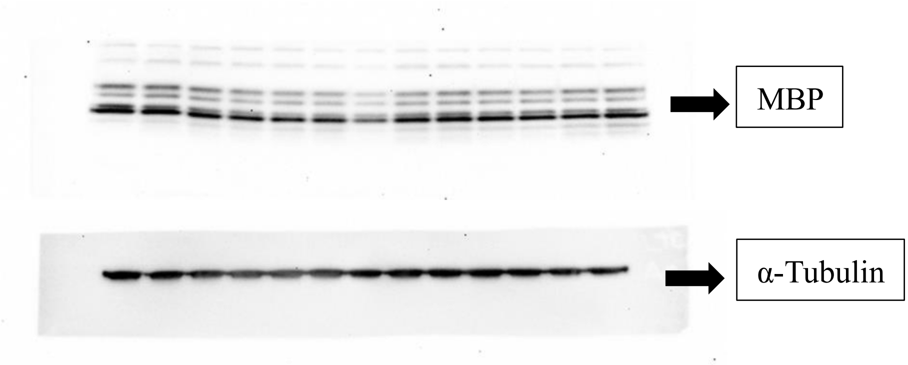

7. Full unedited gel for Figure 5B:

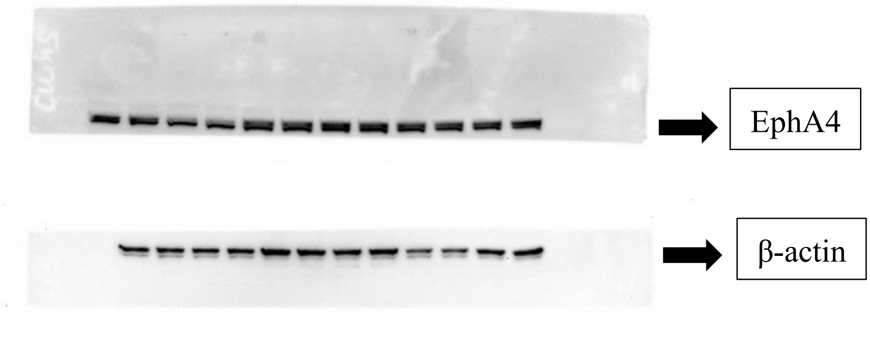

8. Full unedited gel for Figure 5D:

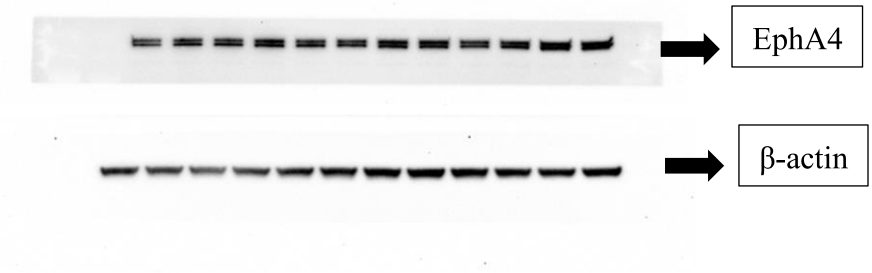

9. Full unedited gel for Figure 5F:

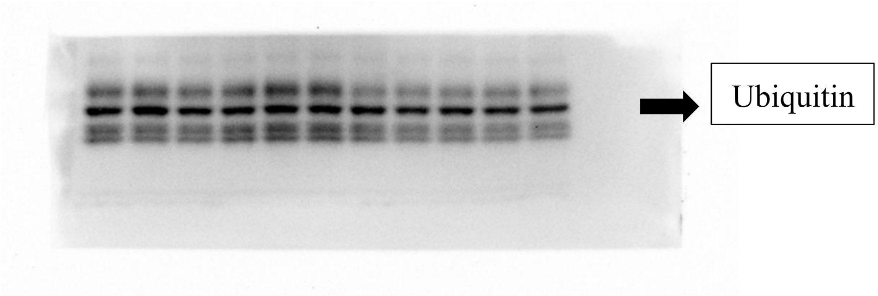

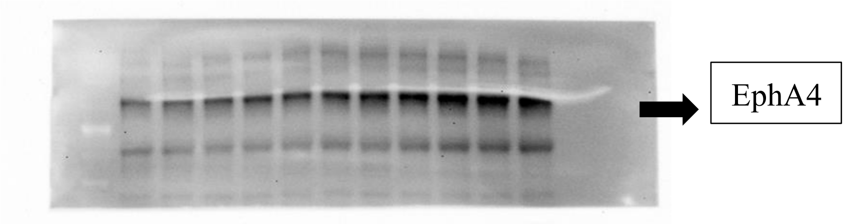

10. Full unedited gel for Figure 6A:

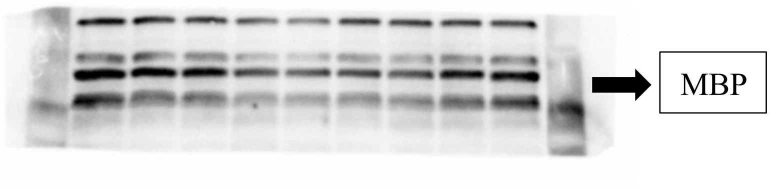

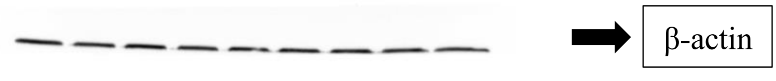

11. Full unedited gel for Figure 6C:

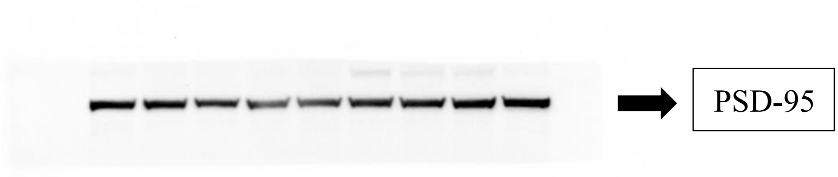

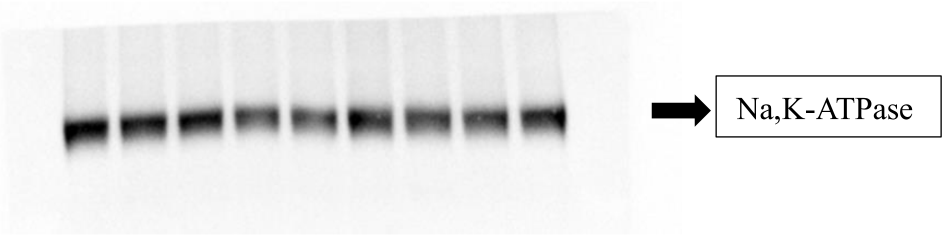

12. Full unedited gel for Figure 7E:

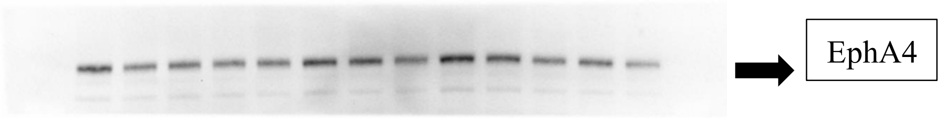

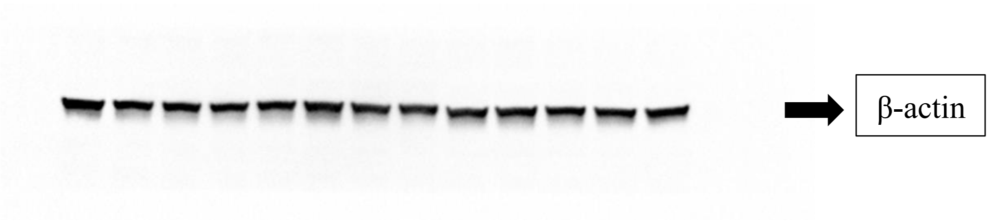

13. Full unedited gel for Figure 7J:

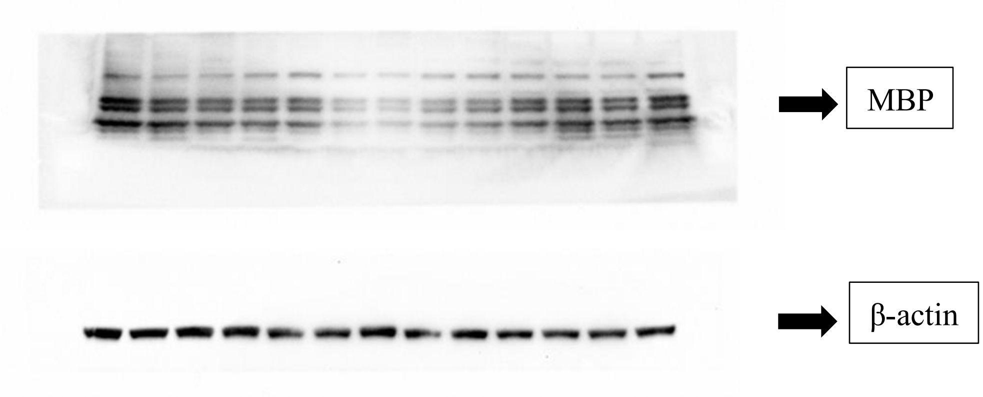

14. Full unedited gel for Figure 8A:

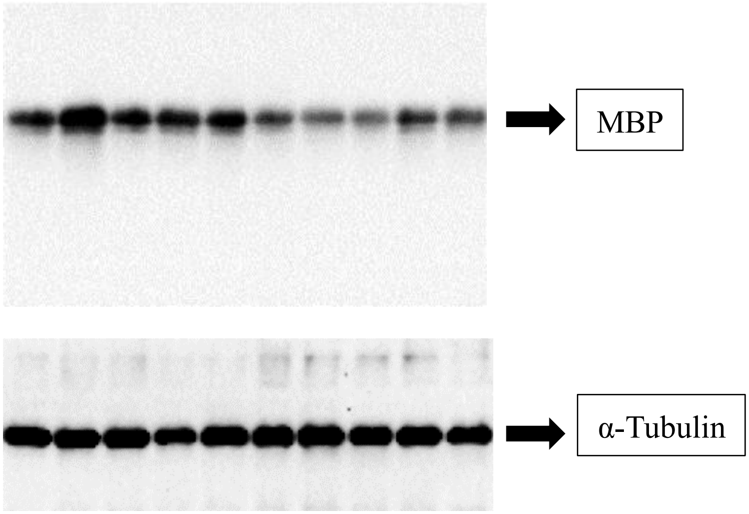

15. Full unedited gel for Figure 8C:

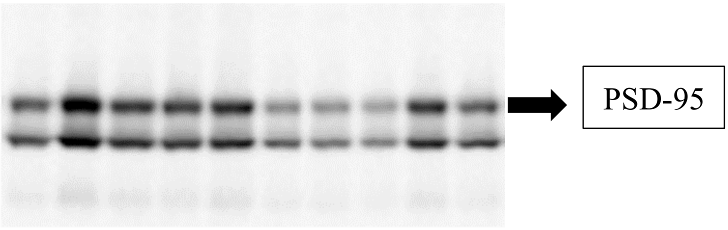

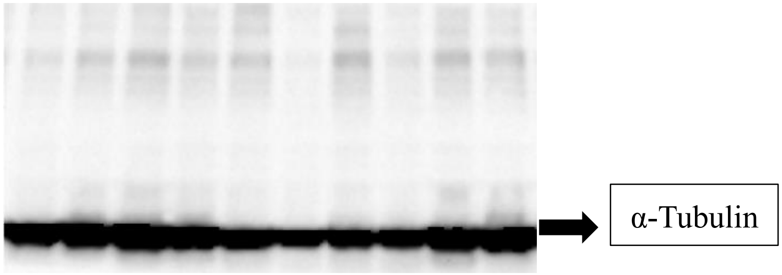

16. Full unedited gel for Figure 8E:

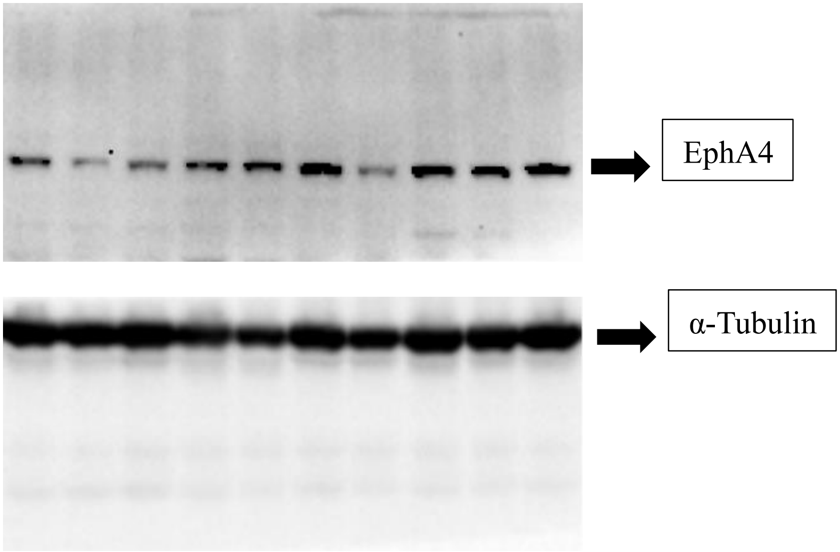

17. Full unedited gel for Figure S2A:

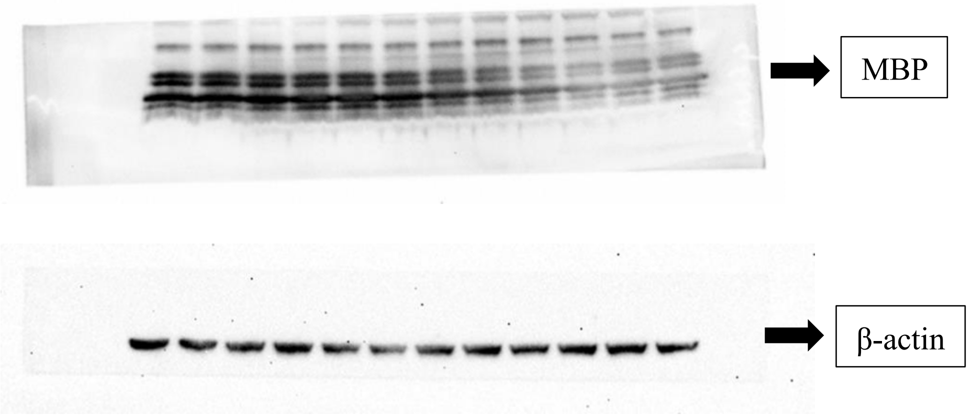

18. Full unedited gel for Figure S2C:

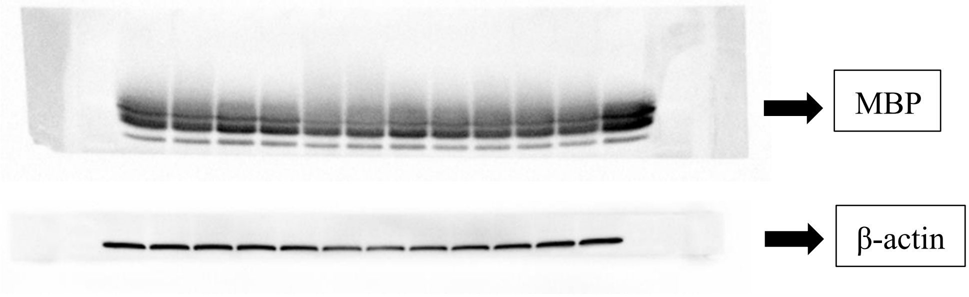

19. Full unedited gel for Figure S5A:

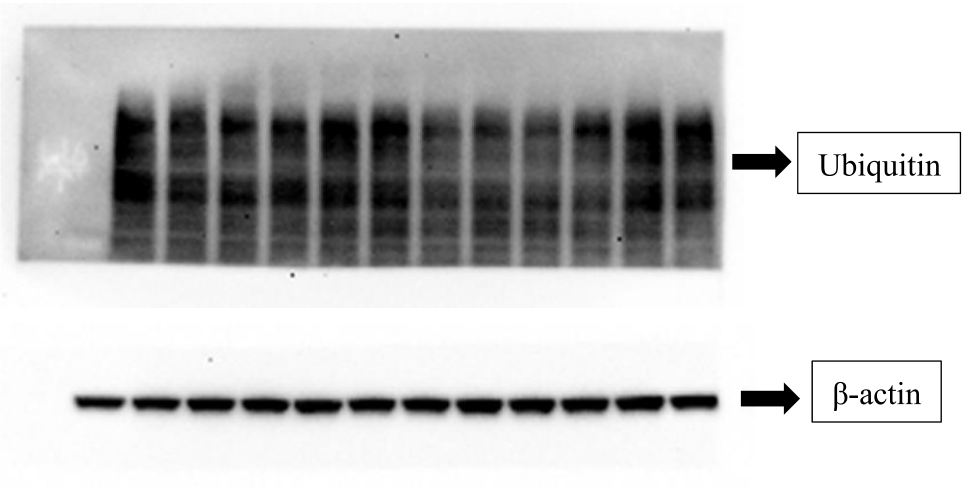

20. Full unedited gel for Figure S5C:

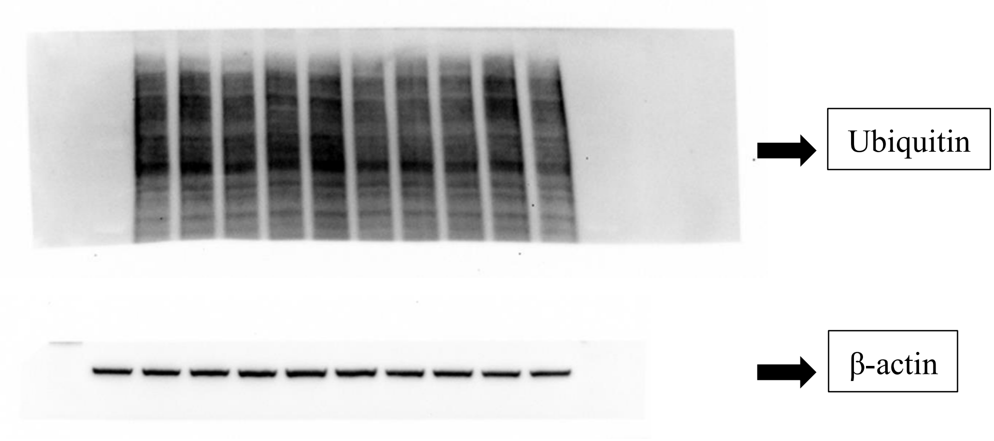

21. Full unedited gel for Figure S6A:

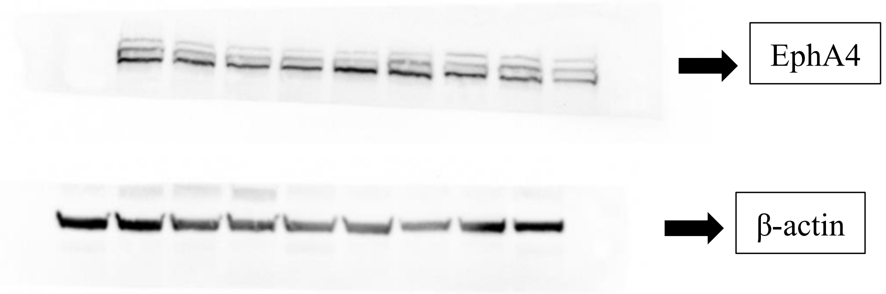

Supplement: Supplemental data [file jci-132-152187-s122.pdf]
